# Genetic Variability among Swine Influenza Viruses in Italy: Data Analysis of the Period 2017–2020

**DOI:** 10.3390/v14010047

**Published:** 2021-12-28

**Authors:** Chiara Chiapponi, Alice Prosperi, Ana Moreno, Laura Baioni, Silvia Faccini, Roberta Manfredi, Irene Zanni, Valentina Gabbi, Irene Calanchi, Alice Fusaro, Maria Serena Beato, Lara Cavicchio, Camilla Torreggiani, Giovanni Loris Alborali, Andrea Luppi

**Affiliations:** 1 OIE Reference Laboratory for Swine Influenza, Istituto Zooprofilattico Sperimentale della Lombardia e dell’Emilia Romagna (IZSLER), 25124 Brescia, Italy; alice.prosperi@izsler.it (A.P.); anamaria.morenomartin@izsler.it (A.M.); laura.baioni@izsler.it (L.B.); silvia.faccini@izsler.it (S.F.); roberta.manfredi@izsler.it (R.M.); irene.zanni@izsler.it (I.Z.); valentina.gabbi@libero.it (V.G.); irene.calanchi@izsler.it (I.C.); camilla.torreggiani@izsler.it (C.T.); giovanni.alborali@izsler.it (G.L.A.); andrea.luppi@izsler.it (A.L.); 2 Biochemistry and Molecular Biology Unit, Department of Life Sciences, University of Parma, 43124 Parma, Italy; 3 Istituto Zooprofilattico Sperimentale delle Venezie (IZSVe), 35020 Legnaro, Italy; afusaro@izsvenezie.it (A.F.); msbeato@izsvenezie.it (M.S.B.); LCavicchio@izsvenezie.it (L.C.)

**Keywords:** influenza A virus, swine, genetic characterization, antigenic characterization, subtyping

## Abstract

Swine play an important role in the ecology of influenza A viruses (IAVs), acting as mixing vessels. Swine (sw) IAVs of H1N1 (including H1N1pdm09), H3N2, and H1N2 subtypes are enzootic in pigs globally, with different geographic distributions. This study investigated the genetic diversity of swIAVs detected during passive surveillance of pig farms in Northern Italy between 2017 and 2020. A total of 672 samples, IAV-positive according to RT-PCR, were subtyped by multiplex RT-PCR. A selection of strains was fully sequenced. High genotypic diversity was detected among the H1N1 and H1N2 strains, while the H3N2 strains showed a stable genetic pattern. The hemagglutinin of the H1Nx swIAVs belonged to HA-1A, HA-1B, and HA-1C lineages. Increasing variability was found in HA-1C strains with the circulation of HA-1C.2, HA-1C.2.1 and HA-1C.2.2 sublineages. Amino acid deletions in the HA-1C receptor binding site were observed and antigenic drift was confirmed. HA-1B strains were mostly represented by the Δ146-147 Italian lineage HA-1B.1.2.2, in combination with the 1990s human-derived NA gene. One antigenic variant cluster in HA-1A strains was identified in 2020. SwIAV circulation in pigs must be monitored continuously since the IAVs’ evolution could generate strains with zoonotic potential.

## 1. Introduction

Swine influenza viruses are a common cause of respiratory disease in pigs. Given their role as a mixing vessel for viruses of human and avian origin, swine are important for the influenza A viruses’ (IAV) ecology [1]. Pigs have, in fact, both human IAV α2,6-galactose (α2,6-Gal)-linked and avian IAV-preferred sialic acid α2,3-Gal-linked receptors [2]. Therefore, swine could be infected by multiple-origin IAV strains and play a role in the generation of potential new pandemic IAV strains, as occurred in 2009 [3].

Moreover, the eight-RNA segmented genome of IAVs contributes to the continuous evolution and reassortment of the circulating strains.

In particular, strains that endemically infect pigs in Europe are the product of mixing events among human, avian, and swine-origin strains [4,5]. The high incidence of IAVs in pigs makes the EU swine population an important reservoir for emerging new strains [6]. The introduction of viral variants into swine, with reverse zoonosis events from humans, and subsequent antigenic evolution has been demonstrated worldwide [7].

Recently, a new H1 nomenclature was proposed to better classify universally H1 lineages: the HA-1A classical lineage, which also includes the H1N1pdm09 lineage, related to the 1918 human influenza pandemic; HA-1B human seasonal lineage; and the HA-1C Eurasian avian lineage [8]. These three major lineages were further divided into second-, third-, and fourth-order clades.

To date, four main subtypes of swIAV circulate in Europe:

- “Avian-like” swine H1N1 (H1avN1, clade HA-1C) lineage, which appeared in 1979 with genes of avian origin and adapted in swine;
- “Human-like” H3N2 lineage, originating from the reassortment of human-like swine H3N2 and a H1avN1 in 1984;
- “Human-like” H1N2 (H1huN2, clade HA-1B) lineage, originating from a human-like H3N2 and a seasonal human H1N1 reassortment in 1994;
- “Pandemic 2009”-origin H1N1 (H1N1pdm09, clade HA-1A), circulating in the swine population since 2009.

At the farm level in Europe, H1avN1 is the dominant subtype (39.2%), followed by other reassortant strains: H1avN2 (12.6%), H1pdm09 subtypes composed of 4.2% H1pdmN1pdm and 4.5% H1pdm09Nx, H1huN2 (11.4%). The H3N2 strains remains at low levels of detection (3.9%) with differences in their circulation at regional level [6,9].

The high incidence of IAV infection in the European swine population and the co-circulation of these different lineages contributed to the generation of reassortant strains with different combinations of swine, avian, and human (seasonal and pandemic) genes [6]. Some of these new strains emerged and replaced the endemic pig swIAV strains; in the UK, the H1N1pdm09 replaced the H1Nx strains; in Denmark, H1avN2 (H1N2dk) supplanted H1huN2 strains [10]; and a new H3huN2 reassortant strain appeared in Danish and German pig herds in 2015/2016 [11,12]. Lineages of H1N1pdm09 strains adapted to swine have been described in several countries [13,14,15]. Moreover, in northern European countries (Germany, Denmark, Sweden), the H1huN2 was replaced by novel reassortant H1pdm09N2 and H1avN2 strains [14], increasing the genetic and antigenic variability described among the HA-1A and HA-1C lineages, cocirculating with different NA genes [5,6,9,16,17,18]. The emergence of these drifted and swine-adapted strains is evidence of the active circulation of swine influenza viruses among European pig herds.

In Italy, we previously described an increasing trend in genetic variability observed in swIAVs isolated in pig farms from 2010 to 2015 with the appearance of H1N1 and H1N2 subtypes, presenting different gene combinations [19]. Moreover, the H1huN2 (i.e., A/swine/Italy/4675/2003, H1clade 1B.1.2.2) subtype, circulating in Italian farms and characterized by a double deletion in the HA1 region and an N2 of seasonal human origin introduced in 2000, has gradually replaced the former European H1huN2 (Scot/94-like) virus [4].

Recently, the presence of pre-pandemic potential features in the European swIAV population has been shown, such as ferret transmissibility and extensive antigenic diversification with low reactivity with human donors’ sera [6]. These factors demonstrate the importance of monitoring circulating swIAV variants and establishing efforts at a national level. In addition, mutations able to confer resistance to MxA, an interferon-induced host restriction factor, were found in the NP gene of both pandemic 2009 and Eurasian avian-like swine IAV lineage [6,20].

In this study, we described the genetic diversification of Italian swIAVs, with particular emphasis on H1 strains, detected in respiratory outbreaks in pig farms between 2017 and 2020.

HA-1A, HA-1B, and HA-1C were detected among H1Nx swIAVs, and the highest variability was found in HA-1C strains with the circulation of imported lineages with high internal gene variability. Three different combinations of amino-acid (aa) deletions in the HA-1C were observed and antigenic drift was confirmed. On the other hand, the HA-1B lineage was still mainly represented by the previously characterized Italian HA-1B.1.2.2-N2 strain [21], showing that this lineage has become well adapted within the pig population. Compared to the 2010–2015 period, we observed an increased circulation of HA-1A among H1 strains, and a new genetic and antigenic cluster was identified in 2020. High genotypic diversity was detected among the H1N1 and H1N2 strains, whilst the H3N2 strains showed a stable genetic pattern.

## 2. Materials and Methods

A total of 9441 samples (nasal swabs or lungs) were collected between 2017 and 2020 in 1533 swine farms affected by respiratory disease and located in Northern Italian regions (Lombardia, Emilia Romagna, Veneto, Piemonte, and Friuli Venezia Giulia) characterized by a high density of pigs. In detail, the samples were delivered for diagnostic purposes to IZSLER (1454 farms, 8714 samples) or IZSVe (79 farms, 727 samples) diagnostic laboratories. Samples were tested in the two aforementioned laboratories with different workflows, as described below.

Viral RNA was extracted from 100 µL of homogenized samples using a One for All vet kit (Indical Bioscience GmbH, Leipzig, Germany) according to manufacturer’s instructions by IZLER. M gene real-time RT-PCR Influenza A virus screening was performed on clinical samples as previously described [22]. Six hundred and seventeen swIAV-positive samples, from independent outbreaks, collected from 397 farms, were sub-typed by IZSLER in order to have a prompt subtype identification by a nested RT-PCR. For this protocol, viral RNA was first amplified with one pair of influenza-specific primers [23] (UNI12 5′GCCGGAGCTCTGCAGATATCAGCRAAAGCAGG3′ and UNI13 5′GCCGGAGCTCTGCAGATATCAGTAGAAACAAGG 3′) at a 10 pmol/μL concentration using SuperScript™ III RT/Platinum™Taq High Fidelity (Thermo Fisher Scientific, Milan, Italy). The IAV RT-PCR included a 2.5 μL RNA template, 0.5 μL forward and 0.5 μL reverse primer, 12.5 μL reaction mix, 0.5 μL SuperScript III RT/Platinum Taq mix, and 8.5 μL RNase free water to obtain a total volume of 25 μL. In this protocol, all influenza segments were amplified simultaneously using one-step RT-PCR and one set of primers adapted to the conserved 3′ and 5′ segment ends. The cycling conditions were as follows: a reverse-transcription step of 30 min at 42 °C, and then denaturation at 94 °C for 2 min, followed by five cycles of 94 °C for 30 s, 45 °C for 30 s and 68 °C for 3 min, followed by additional 30 cycles of 94 °C for 30 s, 57 °C for 30 s and 68 °C for 3 min with a final elongation step at 68 °C for 5 min. The HA and NA subtype was detected using a double-nested PCR using the product of the first universal RT-PCR as a template. Two separated multiplex reactions were set up to amplify HA and NA genes with the HotStarTaq Master Mix Kit (Qiagen, Milan, Italy) using primers listed in Table 1. Each multiplex PCR was performed in a reaction mixture of 25 µL containing 12.5 µL of HotStarTaq Master Mix 2×, 5 µL of H_2_O, 12.5 pmol of each primer H1N2_for, H1N2_rev, H1N1_for, H1N1_rev, N1_for, N1_rev, N2_for, N2_rev, 25pmol of primers SW-H1 FOR 107, SW H1 REV 623, H3_for, H3_rev, and 2.5 µL of DNA product of the universal RT-PCR pre-diluted 1:20. Amplifications were obtained with the following cycling conditions: 15 min at 95 °C of initial denaturation, then 40 cycles at 94 °C for 45 s, annealing at 55 °C for 45 s, and extension at 72 °C for 1 min followed by an extension step at 72 °C for 7 min. PCR amplicons were separated and visualized on 2% agarose gel. Viral subtypes were differentiated according to the length of each amplification product (Table 1) and classified preliminarily as H1N1, H1N2, H3N2, and H1N1pdm09.

Viral RNA was extracted from 100 µL of homogenized samples using a KingFisher™ Flex Purification System (Waltham, MA, USA), a semiautomated method (Thermo Fisher Scientific) with the ID GENE MAG UNIVERSAL EXTRACTION KIT (ID Vet Grabels, France) by IZSVe laboratory. The RT-PCR targeting the M gene, described by Hoffmann et al., 2010 [24], was used for the screening of the samples. Fifty-five positive samples collected during different outbreaks from 55 farms were subtyped by IZSVe by an hemagglutinin- and neuraminidase-specific tetra- and triplex real-time RT-PCR [25].

Both laboratories attempted virus isolation of positive samples, inoculating 11-day-old SPF embryonated chicken eggs or cell cultures (MDCK or CACO-2) [26,27]. A selection of 413 isolated swIAVs or positive diagnostic samples were further analyzed from a genetic and antigenic point of view. Selected swine viruses came from independent respiratory outbreaks; when more than one virus was isolated from the same respiratory outbreak, we selected only one of them for further analysis.

Viral RNA was extracted from isolated viruses or clinical samples as described above and full genome sequencing was performed as previously described by Lycett et al. [28] using SuperScript^®^ III One-Step RT-PCR System with Platinum^®^ Taq High Fidelity (Thermo Fisher Scientific). RT-PCR products were purified with NucleoSpin^®^ Gel and PCR Clean-up (Macherey-Nagel, Carlo Erba, Italy). DNA libraries were made with an NEXTERA-XT kit (Illumina Inc. San Diego, CA, USA) according to the manufacturer’s instructions. Pooled libraries were sequenced on a MiSeq Instrument (Illumina) by using a Miseq Reagent nano Kit v2 in a 150-cycle paired-end run. Data were de novo assembled by the CLC genomic workbench v.11 (Qiagen, Milan, Italy). Sequences of all the strains of the study were submitted to Genbank (accession numbers MW220195 -MW220833_2017, MW170386-MW171247, MW169476- MW170361, MW244100-MW244371, MW621504-MW621857, MZ404272-MZ404287, MZ483984-MZ484071, MZ477531-MZ477743, MZ541572-MZ541601, MZ542323).

All eight gene segments were preliminarily analyzed, performing a nucleotide query step in the Genbank database (http://blast.ncbi.nlm.nih.gov/BLAST; accessed on 21 October 2021) and identifying closely related sequences. European swine IAV reference sequences were retrieved from the Genbank Influenza virus resource database (https://www.ncbi.nlm.nih.gov/genomes/FLU/Database/nph-select.cgi?go=database; accessed on 21 October 2021). Gene sequences were then aligned with ClustalW using MEGAX [29]. For the purposes of lineage assignment, phylogenetic trees of the individual segments were inferred with the maximum likelihood (ML) method implemented in IQ-TREE-2 [30,31,32]. The robustness of the ML trees was statically evaluated by bootstrap analysis with 5000 bootstrap samples. A likelihood mapping analysis was conducted in IQ-TREE2 with 25,000 quartets for each alignment, with the model being automatically selected.

Trees were visualized using Figtree v. 1.4.4 (http://tree.bio.ed.ac.uk/software/figtree accessed on 20 December 2021) or using CLC genomic workbench v.11. The origin of each segment was named by its clustering with reference strains. In addition, swine H1 sequences were analyzed and named using the swine H1 influenza classification tool (http://www.fludb.org; accessed on 21 October 2021) [8].

Due to the segmented genome, each virus had to be described with a specific gene combination, and viruses were further classified into numbered genotypes.

The sequences of the swIAV HA, NA, and NP gene segments were aligned using ClustalW as described above. The predicted amino acid (aa) sequence was obtained for each gene. In addition, in order to analyze the mutations able to confer resistance to MxA, amino acid positions 53, 100, and 313 were analyzed for NP of the H1N1pdm09 lineage [33,34] and positions 48, 98, and 99 for NP of avian origin [6,20].

Selected H1 strains belonging to recently identified genetic clusters were tested in triplicate by HI test using hyperimmune pig sera against the following Italian strains: A/swine/Italy/284922/2009 H1N2 (1B.1.2.2), A/swine/Italy/311368/2013 H1N1 (1C.2.1), A/swine/Italy/311349/2013 H3N2, A/swine/Italy/282866/2013 H1N1 (1A.3.3.2) [27]. These hyperimmune sera exhibited HI titers against the homologous viruses that ranged between 1:640 and 1:2560.

## 3. Results

### 3.1. swIAV Subtypes Circulation over the Four Years (2017–2020)

A total of 1533 pig farms (9441 samples) presenting respiratory problems, located in an area with a high density of pig farms, were examined for the presence of swIAVs by real-time RT-PCR over a four-year period (2017–2020); 676 of them (44%) tested positive at least once with M gene RT-PCR.

During the examined period, 672 swIAVs were subtyped from temporally different outbreaks occurred in different times.

The H1N2 subtype was detected in 45% of the outbreaks, followed by the H1N1 subtype (35%), H3N2 subtype (12%), and H1N1pdm09 (6%), with the sporadic detection of mixed infections with two subtypes (2%). The detection of H1N1 and H1N2 was stable in the study period, while H3N2 detection decreased from 12 to 15% in 2017–2019 to 5% in 2020. On the other hand, the positivity for H1N1pdm09 increased rapidly from 1% in 2017 to 15% in 2020 (Table 2).

### 3.2. Genotypes Analysis

**Figure 1:**
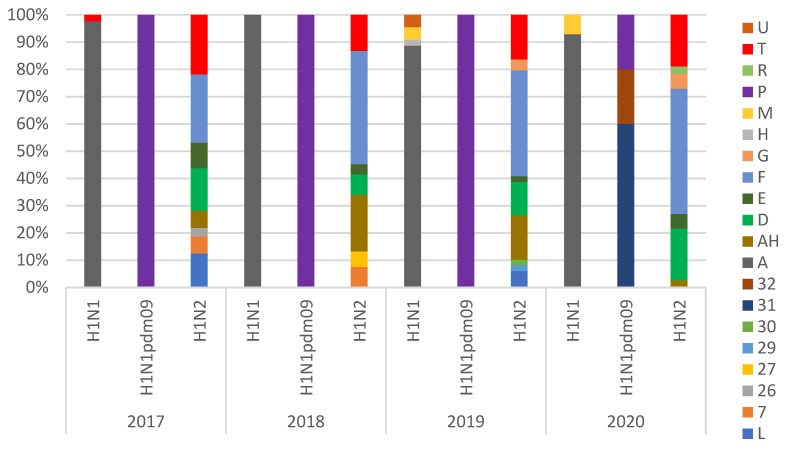
Percentage of genotypic diversity of H1 swIAV Italian strains per year detected between 2017 and 2020. Genotype identification is described in Table 3 and Table 4. Strains were classified in subtypes: H1N1 (HA-1B/HA-1C-N1), H1N1pdm09 (HA-1A-N1), H1N2 (HA-1A/HA-1B/HA-1C-N2).

Using full genome sequencing, we analyzed 358 H1Nx (171 H1N2, 160 H1N1, and 27 H1N1pdm09) and 55 H3N2 swIAV strains (Table 3 and Table 4). A preliminary phylogenetic analysis of the whole genomes of Italian isolates, with the arrangement of the eight genes, allowed us to further assign each virus to a specific lineage, genotype, and origin. H1N2 was the subtype with the highest genotypic variability (with the detection of 18 different gene combinations), followed by H1N1 (HA-1C lineage) with seven genotypes and H1N1pdm09 (HA-1A lineage) with three genotypes. The relative proportion of these genotypes showed the dominance of the endemic strains for H1N2 (HA-1B.1.2.2-ItN2, genotype F) and H1N1 (HA-1C-N1av, genotype A). It is noteworthy that the H1N1pdm09 underwent multiple reassortment events with the identification, in 2020, of two previously undescribed H1N1pdm09-derived genotypes (HA-1A.3.3.2-N1av, genotypes 31 and 32) (Figure 1, Table 4).

**Figure 2:**
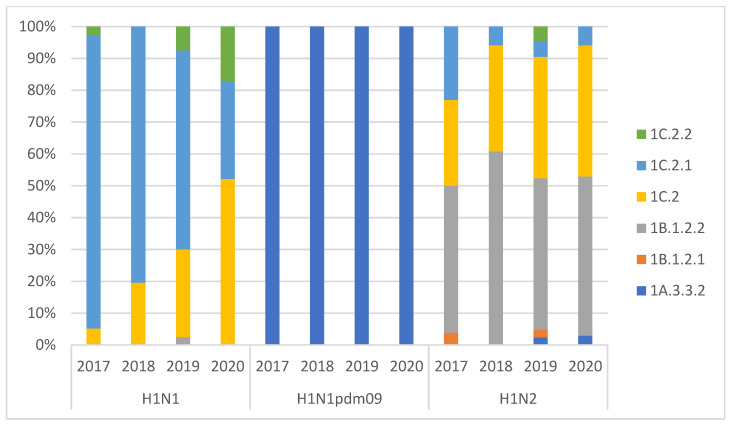
H1 lineage differentiation per year of collection of swIAVs detected. H1 nomenclature was applied as described by Anderson et al. Strains were classified in subtypes: H1N1 (HA-1B/HA-1C-N1), H1N1pdm09 (HA-1A-N1), H1N2 (HA-1A/HA-1B/HA-1C-N2).

HA-1B.1.2.2 was almost the only human-like HA detected in Italy in H1N2 strains together with reassortant strains with HA-1C-N2, which represent, respectively, 38.6% and 50.9% of the identified H1N2 lineages (Figure 2). The H1N1 genotypic diversity increased since 2019, with the detection of several introductions of different genotypes harboring HA-1C.2 in the country (Figure 1 and Figure 2, Table 4).

All of the 55 sequenced H3N2 strains had the same genotype, with HA and NA genes Ghent-84 derived and internal genes of avian origin (Table 3).

### 3.3. Phylogenetic Analysis of Italian swIAVs

**Figure 3:**
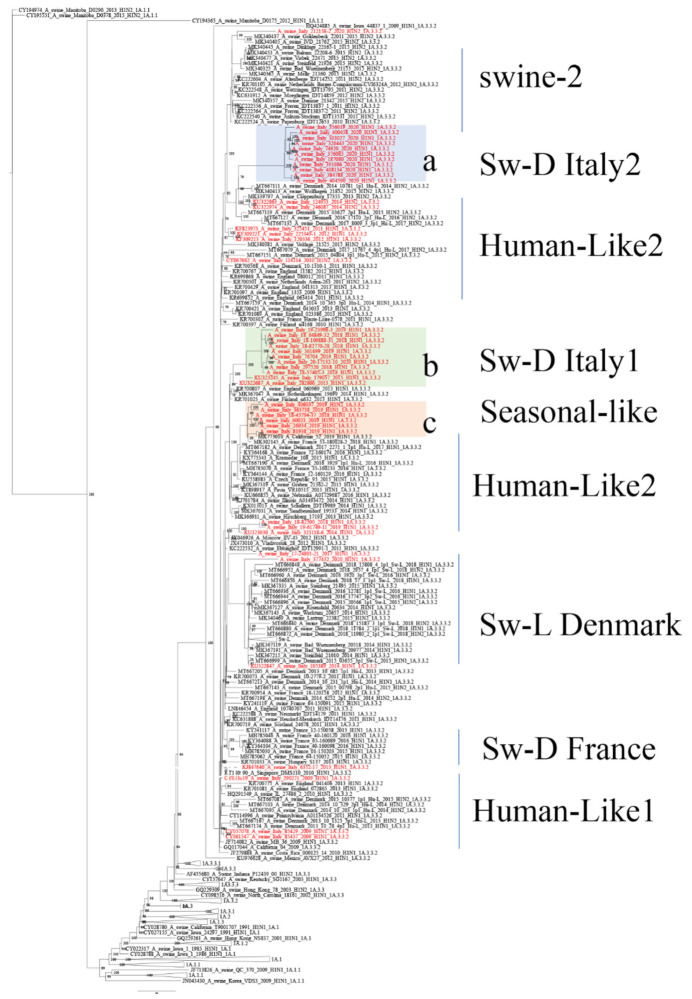
ML phylogenetic tree of 270 HA-H1A with the 29 HA-1A, 1A.3.3.2, sequences (1611 nucleotides) of strains analyzed in this work. Tree is inferred with European reference sequences retrieved from Genbank collected in the years 2009–2020 with the reference sequences used to construct the global swine H1 clade classification reference tree, which is used to classify the clade of the HA/H1 viruses [8]. The three main sub-clusters circulating recently in the country (**a**–**c**) are highlighted in light blue, green, and pink, respectively and the closest HA sequences matching strains in sub-clusters a, b and c are included. Italian sequences are in red;. Bootstrap values under 70% are not shown; scale bar represents number of nucleotide substitutions per nucleotide site. Sequences retrieved from Genbank are identified by their accession number. Clusters are named according to recent papers describing swIAVs in Europe [6,9,18].

**Figure 4:**
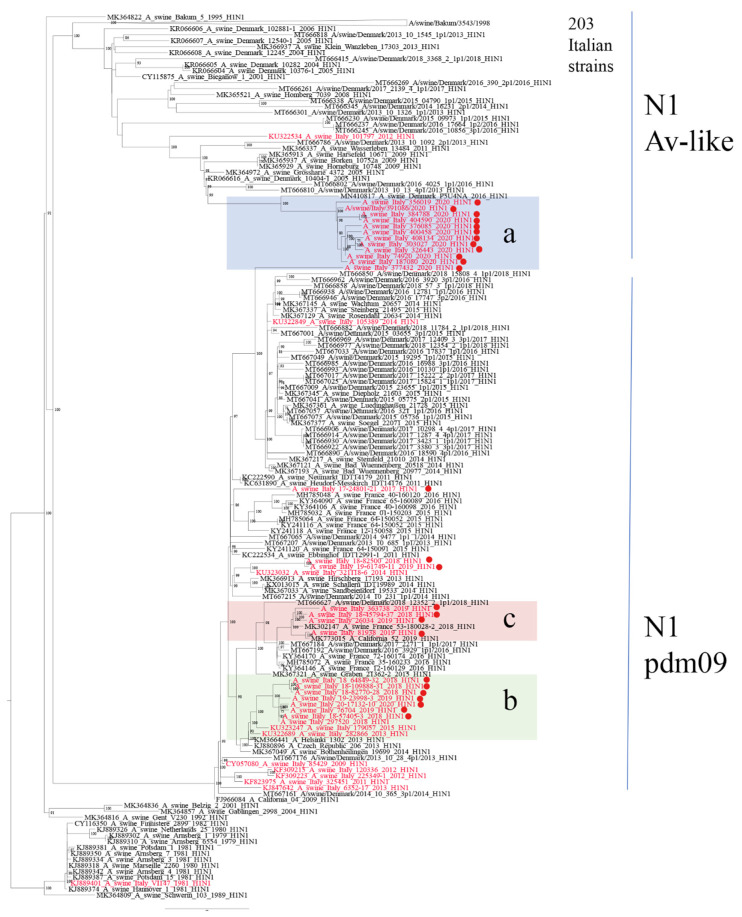
Phylogenetic tree of 199 N1 sequences (1373 nucleotides) of strains sequenced in this work analyzed with 442 representative swine and human N1 sequences, collected from 2009, retrieved from Genbank; the closest NA sequences matching strains in sub-clusters a, b and c are included. Italian sequences are in red with dots highlighting recent strains (2017–2020). The three main sub-cluster (**a**–**c**) are highlighted in light blue, green, and pink, respectively. To simplify visualization, a group of 203 Italian N1 strains related to the Eurasian avian-like N1 gene A_swine_Bakum_3543_1998 have been collapsed into one branch. Bootstrap values under 70% are not shown; scale bar represents number of nucleotide substitutions per nucleotide site. Sequences retrieved from Genbank are identified by their accession number.

HA-1A strains circulating up to 2019 (clade 1A.3.3.2) confirmed the data collected previously [19] with the cocirculation of a group of swine-adapted strains, swine-D Italy1 (sub-cluster b, Figure 3) detected in Italy since 2013 and strains related to seasonal H1pdm09 strains (sub-cluster c, Figure 3). In 2020, we detected, in 11 farms, the circulation of a new variant of H1N1pdm09, swine-D Italy2 (sub-cluster a, Figure 3), whose closest related strains were H1N2 strains detected in Denmark and Germany in the years 2014–2015 (i.e., A/swine/Denmark/10781/2014, A/swine/Wolfaghen/21852/2015). The variant presented two different reassortant patterns: genotype31, characterized by N1 and NS of Eurasian avian-like origin and the remaining internal genes of pdm09 origin, and genotype32, with only N1 Eurasian avian-like origin and all the remaining genes of pdm09 origin (Table 4). All of these 11 strains shared a common N1 gene, clustering apart from the cocirculating avian-like N1 (A/swine/Bakum/3543/1998 H1N1) gene associated to Italian HA-1C strains (Figure 4) and slightly related to strains detected in Denmark and Germany (i.e., A/swine/Denmark/P5U4NA/2016 H1N1). The recent strains, belonging to sub-cluster *b* and *c*, reacted in HI tests (Table 5) with the reference pandemic swine antiserum against strain A/swine/Italy/282866/2013 (sub-cluster b, Figure 3). On the other hand, the new strains of sub-cluster a were antigenically different from HA-1A swIAV strains circulating previously in Italy and did not react with any IZSLER laboratory reference antiserum tested using HI testing (Table 5). Strains related to the A/swine/Italy/290271/2009 (Genotype P, Human-like1 cluster) have not been detected since 2013 in Italian pigs. Two farms tested positive for the HA-1A, introduced from 2012 human seasonal H1N1pdm09 strains, detected since 2014 (i.e., A/swine/Italy/312118/2014, genotype P, Human-like2 cluster). Only two strains showed a relation to the HA-1A antigenic variant circulating in Germany Wachtum/2014-like [16], including the Sw-L strains detected in Denmark recently [18].

**Figure 5:**
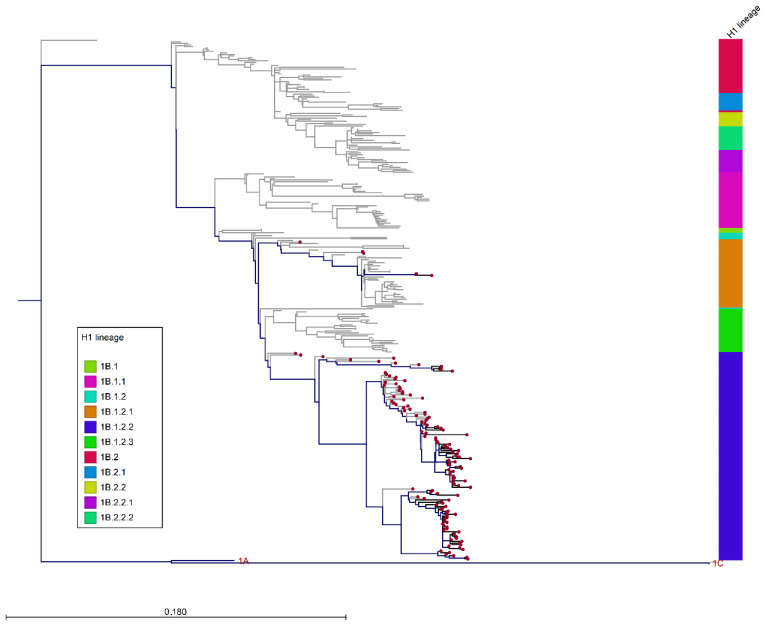
ML phylogenetic tree of 1460 HA-H1 sequences (*n* = 270 HA-1A, *n* = 322 HA-1B, and *n* = 857 HA-1C) with the 86 HA-1B sequences (1611 nucleotides) of strains analyzed in this work inferred with swine references sequences retrieved from Genbank. Scale bar represents number of nucleotide substitutions per nucleotide site. Tree visualized using CLC genomic workbench v.11. To simplify visualization, HA-1A and HA-1C branches have been collapsed. The Italian sequences are represented by red dots; the bar colors distinguish HA-1B clusters. Branch colors distinguish strains collected before 2017 (grey) or after 2017 (black).

The phylogenetic analysis of H1 of human origin HA-1B genes (Figure 5) showed the persistence, in Italy, of the 1B.1.2.2 clade, Δ146–147 variant, previously described [21]. Strains collected after 2017 distributed into two sub-clusters, still circulating, associated with it-N2 among pig farms (Supplementary Figure S1). Sporadic detections of human origin 1B.1.2.1 were identified.

**Figure 6:**
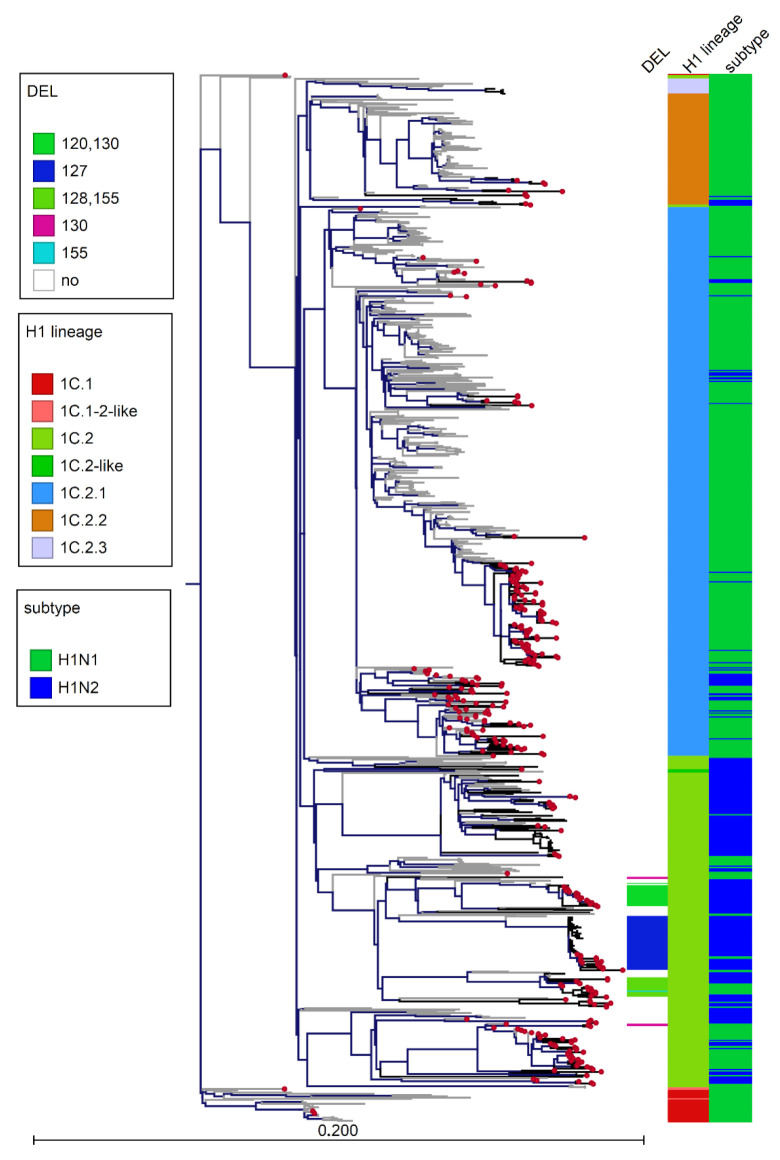
ML phylogenetic tree of 857 HA-1C sequences among which 241 HA-1C were sequences (1611 nucleotides) obtained in this work inferred with European references sequences retrieved from Genbank and collected in the years 2009–2020. The reference sequences used to construct the global swine H1 clade classification reference tree, which is used to classify the clade of the HA/H1 viruses, are included [8]. Moreover, the closest HA sequences matching strains in subclusters are included. Scale bar represents number of nucleotide substitutions per nucleotide site. Tree visualized using CLC genomic workbench v.11. Italian strains are marked by red dots and branch colors distinguish strains isolated before 2017 (grey) and after 2017 (black). Bars on the right panel distinguish the clade and highlight 1C.2 strains with different HA deletions and their amino acid positioning (DEL or NO, no deletion) and virus subtype.

The genetic analysis of the circulating H1avN1 viruses showed an increased variability with respect to the situation described in 2015 [19], since multiple variants of H1C were detected. Different sub-clusters, grouped in the 1C.2 lineage with German and Danish strains, in combination with both N2 and N1 genes, have been identified since 2019 (Figure 6). Moreover, in this lineage, five different patterns of deletion in the receptor binding site region [35] were identified according to H1 numbering [36]: Δ127 (*n* = 13), Δ130 (*n* = 2), Δ120,130 (*n* = 17), Δ128,155 (*n* = 15), and Δ155 (*n* = 1).

Deletions Δ130 and Δ120,130 were present in two strains, clustering with Italian sequences, isolated in Denmark in 2006 (A/swine/Denmark/10547-4/2006 H1N1) and 2016 (A/swine/Denmark/AR8888/2016), respectively (Supplementary Figure S3), and the deletion Δ127 was present in Danish strains detected in 2017. Representative strains for each of the Δ120,130; Δ128,155; Δ127 were tested by HI test using Italian reference antiserum A/swine/Italy/311368/2013, lineage 1C.2.1, with no reaction (Table 5). The complete, non-collapsed, phylogenetic H1 tree is available in Supplementary Figure S3.

H3N2 strains showed no significative reassortant patterns, all HA genes belonged to the H3(84) lineage and no H3huN2 variant was detected in the examined period (data not shown).

The NP gene was analyzed globally in the period considered after the alignment of 375 sequences (1441 nucleotides). Translated sequences were aligned; NP aa positions 48, 53, 98, 99, 100, and 313 were compared. MxA resistance mutations 48Q, 98K, and 99K (for avian-origin NP) and 53D, 100I, 313V (for pandemic-origin NP) were recorded. Among avian-origin NP genes (n. 298), 98% harbored the complete set of mutations, 48Q, 98K, and 99K, able to confer resistance to mammal host MxA, and 1.3% harbored at least one mutation (48Q). The analysis of NP genes (*n* = 77) of pandemic origin showed the presence of both 100I and 313V mutations in 87% of strains, whilst only 0.9% of the strains possessed three mutations: 53D, 100I, 313V. The 53D mutation was reported to be lost when pandemic-origin NP was reintroduced in swine, and this loss was a sign of adaptation to swine [33]. In all the swIAVs, the 313V mutation was present.

## 4. Discussion

The detection of swIAV subtypes in Italy between 2017 and 2020 showed an evolving situation that deserves continuous monitoring. H1avN1 and H1N2 were the most frequently detected subtypes in Italian swine farms. In 2020, we observed a decreased circulation of H3N2 virus strains and an increased detection of H1-1A viruses accompanied by an increased variability among HA-1A genotypes (Figure 1). We demonstrated the circulation in Italy of three HA-1A3.3.2 sub-clusters: a and b (both swine-adapted) and c, seasonal human-derived. In particular, strains belonging to cluster *a* were shown to be new strains circulating in the country of previously unreported genotypes, detected in eleven farms. The HA and NA genes of these strains clustered with human-derived swine H1-1AN2 strains detected in Denmark and Germany since 2013 (so-called Hu-L strains [18]) and with the H1N1 recently identified in Germany and Denmark, respectively, suggesting they could be the product of reassortments of viruses introduced by pigs imported from central and northern Europe. These reassortant viruses showed different combinations of internal genes (pdm09 and avian-origin) and have been demonstrated to be antigenically different to the previously circulating HA-1A in strains Italy (Table 5). The fitness of these genotypes, provisionally named genotype31 and genotype32, should be further evaluated. In the first years after the 2009 pandemic, H1N1pdm09 did not replace the H1avN1 strains through farm-to-farm transmission in European and Italian pigs, probably because of their cross-reactivity, which conferred cross-protective immunity to the animals. On the other hand, as previously shown in Germany [16], antigenic variants of HA-1A strains (e.g., Papenburg/2010-like or Wachtum/2014-like viruses) could spread with persistent infections in pigs.

The H1N2 subtype was confirmed to be the most variable subtype due to the circulation in Italy of six lineages of H1 gene (1C.2, 1C.2.1, 1C.2.2, 1B.1.2.1, 1B.1.2.2, 1A.3.3.2) in combination with three N2 lineages (it-N2, N2g, N2s) and pandemic- or avian-origin internal genes. During the study period, eighteen H1N2 genotypes were detected. The genotype F (1B.1.2.2-itN2) was the most prevalent strain circulating among the Italian farms (38.6%), swine-endemic Italian H1N2 remaining the most well-adapted. Moreover, the reassortant avian HA-1C.2-N2g strains, probably introduced with live animals (growing pigs and gilts of parental lines) from countries of central/northern Europe (Germany and Denmark), were frequently detected (Table 3, Figure 6). In particular, the high number of combinations of HA-1C genes with different lineages of NA (both N1 and N2 genes) enhanced variability, showing the particular fitness of these reassortant strains to the pig population. This variability was increased by the detection of new variants of HA-1C.2, with deletions in the receptor binding site region and with different antigenic properties that need to be investigated and monitored in the future. The finding, in public sequence databases, of swIAV Danish sequences clustering with the Italian deleted HA-1C.2 strains with deletion Δ127, Δ130, and Δ120,130 supported the hypothesis of the introduction in Italy of these variants through the importation of live animals. The detection of these variants suggests the need to update HA-1C nomenclature, since many strains, grouping in different sub-clusters, have been provisionally categorized in lineage 1C.2.

Italy imports 50,000 tons of live animals per year (data 2020), mainly from Denmark, the Netherlands, and Germany [37], with an irrelevant role as a swine exporter. The circulation of endemic viruses and the continuous introductions of different viral strains from central and northern Europe makes Italy an important hotspot for the emergence of new genotypes unique to this area. As a consequence, distinct genotype frequencies and genetic variability distinguish Italy from other European countries [6,14,16,18,20,33].

The evidence of the circulation of pandemic-origin genes adapted to swine and the detection of MxA-resistance mutations in NP genes of avian and pandemic origin in the Italian strains analyzed in this study confirmed the significance of the role of swine as an intermediate host. The study’s results highlight the importance of systematic surveillance against IVs in the swine population, which must be constantly updated in order to be able to dynamically control the viral hypervariability. Such surveillance is essential in order to have an early warning system toward the next potential pandemic [20,33]. Moreover, the cocirculation of antigenically variant viruses of each of the H1N1 and H1N2 subtypes needs to be carefully considered using reference viral strains with antibody screening tests such as HI testing from pig samples.

## Figures and Tables

**Table 1:** List of primers used in the nested multiplex PCR for virus subtyping and PCR product expected size.

Primer Name	Sequence 5′-3′	Subtype-Ref	PCR Product Expected Size
H1N2_for (H1-1B)	GCTACCATGCGAACAATTCA	H1 [19]	241
H1N2_rev (H1-1B)	TCAGCATTTGGTGTTTCTGC		
H1N1_for (H1-1C)	CTGCACTGAAAGCTGACACC	H1 [19]	327
H1N1_rev (H1-1C)	GCTGCTCCCTTAATTCCTCA		
SW-H1 FOR 107 (H1-1A)	CAGACACTGTAGACACAGTAC	H1pdm09 [20]	516
SW H1 REV 623 (H1-1A)	CTAGTAGATGGATGGTGAATGC		
H3_for	CARATTGARGTGACHAATGC	H3 [19]	722
H3_rev	GGTGCATCTGAYCTCATTA		
N1_for	TGAAATACAATGGCATAATAAC	N1 [19]	514
N1_rev	GGATCCCAAATCATCTCAAA		
N2_for	GGAAAAGCATGGCTGCAT	N2 [19]	791
N2_rev	GTGCCACAAAACACAACAAT		
R = A/G, Y = C/T, D = G/A/T, H = A/C/T.			

**Table 2:** swIAV subtype positivity per year considered. Strains were classified in subtypes: H1N1 (H1-1B/H1-1C-N1), H1N1pdm09 (H1-1A-N1), H1N2 (H1-1A/H1-1B/H1-1C-N2).

	*n*. of Subtyped Outbreaks	H1N1	H1N2	H3N2	H3N1	H1N1 pdm09	Mixed Infection
2017	196	41%	41%	15%	0%	1%	2%
2018	164	36%	48%	12%	1%	3%	1%
2019	149	31%	44%	17%	1%	5%	2%
2020	163	31%	47%	5%	0%	15%	3%
2017–2020	672	35%	45%	12%	0%	6%	2%

**Table 3:** List of genotypes detected among 171 analyzed H1N2 strains and 55 H3N2 and relative percentages. Genotype nomenclatures assigned as described previously [5,6]. Undescribed genotypes (*) are numbered arbitrarily [19]. H1-clade are classified according to [8], and H3(84) correspond to Ghent-like. NA lineages assigned as described in [5,6] (N2g Ghent-like; N2s, Scotland-like; It-N2; A/swine/Italy/4675/2003).

Subtype	Nomenclature	HA	NA	PB2	PB1	PA	NP	M	NS	%
H1N2	F	1B.1.2.2	It-N2	av	av	av	av	av	av	38.6%
	AH	1C.2	N2g	pdm	pdm	pdm	pdm	pdm	av	12.9%
	T	1C.2.1	N2g	pdm	pdm	pdm	pdm	pdm	pdm	0.6%
		1C.2	N2g	pdm	pdm	pdm	pdm	pdm	pdm	17.5%
	L	1C.2.1	It-N2	av	av	av	av	av	av	1.8%
		1C.2	It-N2	av	av	av	av	av	av	2.3%
	D	1C.2.1	N2g	av	av	av	av	av	av	5.8%
		1C.2	N2g	av	av	av	av	av	av	7.0%
	E	1B.1.2.1	N2g	av	av	av	av	av	av	1.2%
		1B.1.2.2	N2g	av	av	av	av	av	av	2.3%
	7 *	1B.1.2.2	It-N2	pdm	pdm	pdm	pdm	pdm	pdm	3.5%
	26 *	1C.2	N2g	pdm	pdm	pdm	pdm	pdm	av	0.6%
	27 *	1B.1.2.2	It-N2	av	av	av	av	pdm	av	1.8%
	29 *	1B.1.2.2	It-N2	av	av	av	pdm	pdm	av	0.6%
	30 *	1A.3.3.2	It-N2	av	av	av	av	av	av	0.6%
	G	1C.2.2	N2s	av	av	av	av	av	av	1.2%
		1C.2	N2s	av	av	av	av	av	av	1.2%
	R	1A.3.3.2	N2g	pdm	pdm	pdm	pdm	pdm	pdm	0.6%
H3N2		H3(84)	N2g	av	av	av	av	av	av	100%

**Table 4:** List of H1N1 detected among 187 analyzed strains and relative percentages among the subtype (H1avN1, 159 samples, H1pdm09N1, 27 samples and H1huN1, 1 sample). The genotype nomenclatures are assigned as described previously [5,6]. Undescribed genotypes (*) are numbered arbitrarily [19]. H1 clades are classified according to [8]. NA lineages are assigned as described in [5]; N1av: Eurasian avian-like; pdm: N1pdm09.

Subtype	Nomenclature	HA	NA	PB2	PB1	PA	NP	M	NS	%
H1N1	A	1C.2.1	av	av	av	av	av	av	av	58.3%
		1C.2	av	av	av	av	av	av	av	19.3%
		1C.2.2	av	av	av	av	av	av	av	4.3%
	M	1C.2.1	av	av	av	av	av	pdm	av	0.5%
		1C.2	av	av	av	av	av	pdm	av	1.6%
	U	1C.2.1	av	pdm	pdm	pdm	pdm	pdm	pdm	1.1%
	P	1A.3.3.2	pdm	pdm	pdm	pdm	pdm	pdm	pdm	8.6%
	31 *	1A.3.3.2	av	pdm	pdm	pdm	pdm	pdm	av	4.3%
	32 *	1A.3.3.2	av	pdm	pdm	pdm	pdm	pdm	pdm	1.6%
	H	1B.1.2.2	av	av	av	av	av	av	av	0.5%

**Table 5:** HI titers of selected strains using Italian pig hyperimmune sera produced against representative Italian 1B.1.2.2, 1C2.1, H3, and 1A.3.3.2 swIAV strains. These hyperimmune sera exhibit HI titers against homologous viruses that ranged between 1:640 and 1:5120.

HI TEST RESULTS					
VIRUS SERUM		A/Swine/Italy/ 284922/2009 H1N2	A/Swine/Italy/ 311368/2013 H1N1	A/Swine/Italy/ 311349/2013 H3N2	A/Swine/Italy/ 282866/2013 H1N1
	LINEAGE	1B.1.2.2	1C.2.1	H3	1A.3.3.2
A/swine/Italy/284922/2009 H1N2	1B.1.2.2	640	<20	<20	<20
A/swine/Italy/311368/2013 H1N1	1C.2.1	<20	1280	<20	<20
A/swine/Italy/311349/2013 H3N2	H3	<20	<20	640	<20
A/swine/Italy/282866/2013 H1N1 sub-cluster (b)	1A.3.3.2	<20	<20	<20	5120
A/swine/Italy/326417/2020 H1N1	1C.2 del 128,155	<20	<20	<20	<20
A/swine/Italy/340406/2020 H1N2	1C.2 del 127	<20	20	<20	<20
A/swine/Italy/381442/2020 H1N2	1C.2 del 128,155	<20	<20	<20	<20
A/swine/Italy/37307/2020 H1N2	1C.2 del 120,130	<20	20	<20	<20
A/swine/Italy/31684/2020 H1N2	1C.2.1	<20	2560	<20	<20
A/swine/Italy/30190/2020 H1N2	1C.2	<20	320	<20	160
A/swine/Italy/187080/2020 H1N1 sub-cluster (a)	1A.3.3.2	<20	<20	<20	<20
A/swine/Italy/297520/2018 H1N1 sub-cluster (b)	1A.3.3.2	<20	20	<20	640
A/swine/Italy/60023/2019 H1N1 sub-cluster (c)	1A.3.3.2	<20	320	<20	5120

## Data Availability

The sequences of the strains of this study are available in Genbank, accession numbers: MW220195 -MW220833_2017, MW170386-MW171247, MW169476- MW170361, MW244100-MW244371, MW621504-MW621857, MZ404272-MZ404287, MZ483984-MZ484071, MZ477531-MZ477743, MZ541572-MZ541601, MZ542323. The sequences dataset used in this study are available in Supplementary File S2.

## Supplementary Materials

The following are available online at https://www.mdpi.com/article/10.3390/v14010047/s1: Figure S1: Phylogenetic tree of N2 sequences. Supplementary File S2: dataset of sequences used in this study. Figure S3: Phylogenetic tree of H1 sequences.
