# Supplementary figures and images for: Genetic Variability among Swine Influenza Viruses in Italy: Data Analysis of the Period 2017–2020
Source: Viruses. 2021 Dec 28;14(1):47. doi: 10.3390/v14010047 (PMC8781872; doi:10.3390/v14010047)

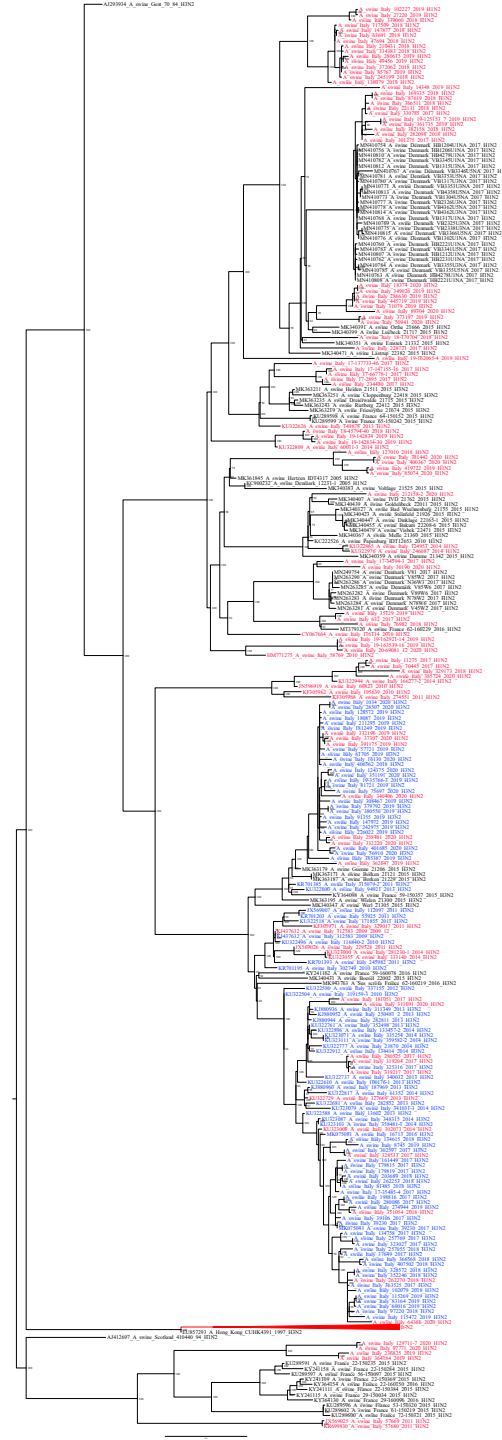

N2g

It-N2

N2s

Supplement: Supplementary file 1 [file viruses-14-00047-s001.zip › Figure_S1_revised.pdf.pdf]
