# Supplementary material for: Genetic Variability among Swine Influenza Viruses in Italy: Data Analysis of the Period 2017–2020
Source: Viruses. 2021 Dec 28;14(1):47. doi: 10.3390/v14010047 (PMC8781872; doi:10.3390/v14010047)
Supplement: Supplementary file 1 [file Figure_S3.pdf › Figure_S3.pdf]

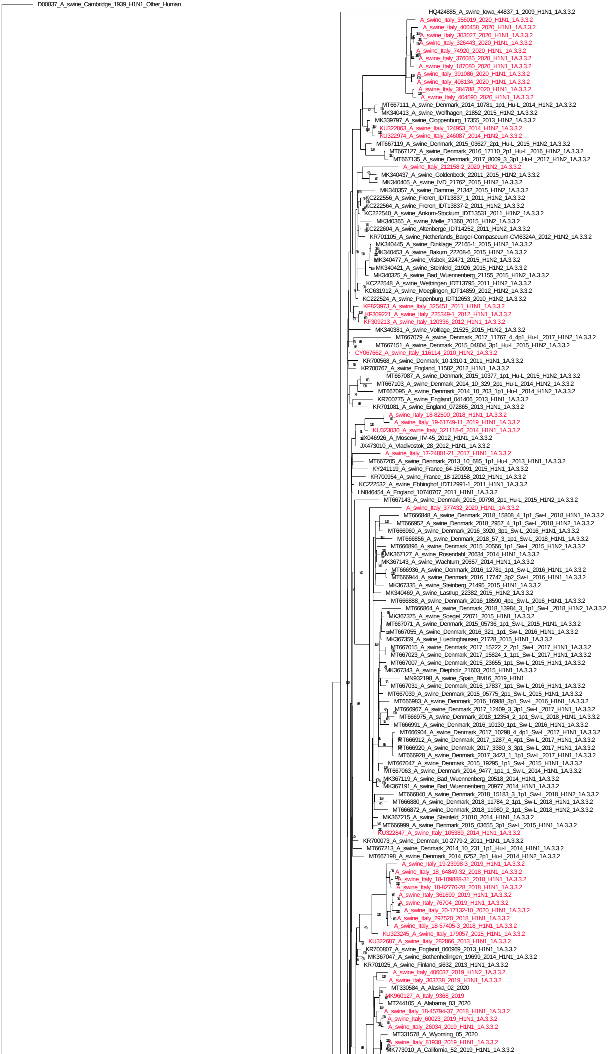
D00837_A_swine_Cambridge_1939_H1N1_Other_Human
HQ424885_A_swine_Iowa_44837_1_2009_H1N1_1A.3.3.2
A_swine_Italy_356019_2020_H1N1_1A.3.3.2
A_swine_Italy_400458_2020_H1N1_1A.3.3.2
A_swine_Italy_303027_2020_H1N1_1A.3.3.2
A_swine_Italy_326443_2020_H1N1_1A.3.3.2
A_swine_Italy_74920_2020_H1N1_1A.3.3.2
A_swine_Italy_376085_2020_H1N1_1A.3.3.2
A_swine_Italy_187080_2020_H1N1_1A.3.3.2
A_swine_Italy_391086_2020_H1N1_1A.3.3.2
A_swine_Italy_408134_2020_H1N1_1A.3.3.2
A_swine_Italy_384788_2020_H1N1_1A.3.3.2
A_swine_Italy_404590_2020_H1N1_1A.3.3.2
MT667111_A_swine_Denmark_2014_10781_1p1_Hu-L_2014_H1N2_1A.3.3.2
MK340413_A_swine_Wolfhagen_21852_2015_H1N2_1A.3.3.2
MK339797_A_swine_Cloppenburg_17355_2013_H1N2_1A.3.3.2
KU322863_A_swine_Italy_124953_2014_H1N2_1A.3.3.2
KU322974_A_swine_Italy_246087_2014_H1N2_1A.3.3.2
MT667119_A_swine_Denmark_2015_03627_2p1_Hu-L_2015_H1N2_1A.3.3.2
MT667127_A_swine_Denmark_2016_17110_2p1_Hu-L_2016_H1N2_1A.3.3.2
MT667135_A_swine_Denmark_2017_8009_3_3p1_Hu-L_2017_H1N2_1A.3.3.2
A_swine_Italy_212158-2_2020_H1N2_1A.3.3.2
MK340437_A_swine_Goldenbeck_22011_2015_H1N2_1A.3.3.2
MK340405_A_swine_IVD_21762_2015_H1N2_1A.3.3.2
MK340357_A_swine_Damme_21342_2015_H1N2_1A.3.3.2
KC222556_A_swine_Freren_IDT13837_1_2011_H1N2_1A.3.3.2
KC222564_A_swine_Freren_IDT13837-2_2011_H1N2_1A.3.3.2
KC222540_A_swine_Ankum-Stockum_IDT13531_2011_H1N2_1A.3.3.2
MK340365_A_swine_Melle_21360_2015_H1N2_1A.3.3.2
KC222604_A_swine_Altenberge_IDT14252_2011_H1N2_1A.3.3.2
KR701105_A_swine_Netherlands_Barger-Compascuum-CVI6324A_2012_H1N2_1A.3.3.2
MK340445_A_swine_Dinklage_22165-1_2015_H1N2_1A.3.3.2
MK340453_A_swine_Bakum_22208-6_2015_H1N2_1A.3.3.2
MK340477_A_swine_Visbek_22471_2015_H1N2_1A.3.3.2
MK340421_A_swine_Steinfeld_21926_2015_H1N2_1A.3.3.2
MK340325_A_swine_Bad_Wuennenberg_21155_2015_H1N2_1A.3.3.2
KC222548_A_swine_Wettringen_IDT13795_2011_H1N2_1A.3.3.2
KC631912_A_swine_Moeglingen_IDT14859_2012_H1N2_1A.3.3.2
KC222524_A_swine_Papenburg_IDT12653_2010_H1N2_1A.3.3.2
KF823973_A_swine_Italy_325451_2011_H1N1_1A.3.3.2
KF309221_A_swine_Italy_225349-1_2012_H1N1_1A.3.3.2
KF309213_A_swine_Italy_120336_2012_H1N1_1A.3.3.2
MK340381_A_swine_Voltlage_21525_2015_H1N2_1A.3.3.2
MT667079_A_swine_Denmark_2017_11767_4_4p1_Hu-L_2017_H1N2_1A.3.3.2
MT667151_A_swine_Denmark_2015_04804_3p1_Hu-L_2015_H1N2_1A.3.3.2
CY067662_A_swine_Italy_116114_2010_H1N2_1A.3.3.2
KR700568_A_swine_Denmark_10-1310-1_2011_H1N1_1A.3.3.2
KR700767_A_swine_England_11582_2012_H1N1_1A.3.3.2
MT667087_A_swine_Denmark_2015_10377_1p1_Hu-L_2015_H1N2_1A.3.3.2
MT667103_A_swine_Denmark_2014_10_329_2p1_Hu-L_2014_H1N2_1A.3.3.2
MT667095_A_swine_Denmark_2014_10_203_1p1_Hu-L_2014_H1N2_1A.3.3.2
KR700775_A_swine_England_041406_2013_H1N1_1A.3.3.2
KR701081_A_swine_England_072865_2013_H1N1_1A.3.3.2
A_swine_Italy_18-82500_2018_H1N1_1A.3.3.2
A_swine_Italy_19-61749-11_2019_H1N1_1A.3.3.2
KU323030_A_swine_Italy_321118-6_2014_H1N1_1A.3.3.2
JX046926_A_Moscow_IIV-45_2012_H1N1_1A.3.3.2
JX473010_A_Vladivostok_28_2012_H1N1_1A.3.3.2
A_swine_Italy_17-24801-21_2017_H1N1_1A.3.3.2
MT667205_A_swine_Denmark_2013_10_685_1p1_Hu-L_2013_H1N1_1A.3.3.2
KY241119_A_swine_France_64-150091_2015_H1N1_1A.3.3.2
KR700954_A_swine_France_18-120158_2012_H1N1_1A.3.3.2
KC222532_A_swine_Ebbinghof_IDT12991-1_2011_H1N1_1A.3.3.2
LN846454_A_England_10740707_2011_H1N1_1A.3.3.2
MT667143_A_swine_Denmark_2015_00798_2p1_Hu-L_2015_H1N2_1A.3.3.2
A_swine_Italy_377432_2020_H1N1_1A.3.3.2
MT666848_A_swine_Denmark_2018_15808_4_1p1_Sw-L_2018_H1N1_1A.3.3.2
MT666952_A_swine_Denmark_2018_2957_4_1p1_Sw-L_2018_H1N2_1A.3.3.2
MT666960_A_swine_Denmark_2016_3920_3p1_Sw-L_2016_H1N1_1A.3.3.2
MT666856_A_swine_Denmark_2018_57_3_1p1_Sw-L_2018_H1N1_1A.3.3.2
MT666896_A_swine_Denmark_2015_20566_1p1_Sw-L_2015_H1N2_1A.3.3.2
MK367127_A_swine_Rosendahl_20634_2014_H1N1_1A.3.3.2
MK367143_A_swine_Wachtum_20657_2014_H1N1_1A.3.3.2
MT666936_A_swine_Denmark_2016_12781_1p1_Sw-L_2016_H1N1_1A.3.3.2
MT666944_A_swine_Denmark_2016_17747_3p2_Sw-L_2016_H1N1_1A.3.3.2
MK367335_A_swine_Steinberg_21495_2015_H1N1_1A.3.3.2
MK340469_A_swine_Lastrup_22382_2015_H1N2_1A.3.3.2
MT666888_A_swine_Denmark_2016_18590_4p1_Sw-L_2016_H1N1_1A.3.3.2
MT666864_A_swine_Denmark_2018_13984_3_1p1_Sw-L_2018_H1N2_1A.3.3.2
MK367375_A_swine_Soegel_22071_2015_H1N1_1A.3.3.2
MT667071_A_swine_Denmark_2015_05736_1p1_Sw-L_2015_H1N1_1A.3.3.2
MT667055_A_swine_Denmark_2016_321_1p1_Sw-L_2016_H1N1_1A.3.3.2
MK367359_A_swine_Luedinghausen_21728_2015_H1N1_1A.3.3.2
MT667015_A_swine_Denmark_2017_15222_2_2p1_Sw-L_2017_H1N1_1A.3.3.2
MT667023_A_swine_Denmark_2017_15824_1_1p1_Sw-L_2017_H1N1_1A.3.3.2
MT667007_A_swine_Denmark_2015_23655_1p1_Sw-L_2015_H1N1_1A.3.3.2
MK367343_A_swine_Diepholz_21603_2015_H1N1_1A.3.3.2
MN932198_A_swine_Spain_BM16_2019_H1N1
MT667031_A_swine_Denmark_2016_17837_1p1_Sw-L_2016_H1N1_1A.3.3.2
MT667039_A_swine_Denmark_2015_05775_2p1_Sw-L_2015_H1N1_1A.3.3.2
MT666983_A_swine_Denmark_2016_16988_3p1_Sw-L_2016_H1N1_1A.3.3.2
MT666967_A_swine_Denmark_2017_12409_3_3p1_Sw-L_2017_H1N1_1A.3.3.2
MT666975_A_swine_Denmark_2018_12354_2_1p1_Sw-L_2018_H1N1_1A.3.3.2
MT666991_A_swine_Denmark_2016_10130_1p1_Sw-L_2016_H1N1_1A.3.3.2
MT666904_A_swine_Denmark_2017_10298_4_4p1_Sw-L_2017_H1N1_1A.3.3.2
MT666912_A_swine_Denmark_2017_1287_4_4p1_Sw-L_2017_H1N1_1A.3.3.2
MT666920_A_swine_Denmark_2017_3380_3_3p1_Sw-L_2017_H1N1_1A.3.3.2
MT666928_A_swine_Denmark_2017_3423_1_1p1_Sw-L_2017_H1N1_1A.3.3.2
MT667047_A_swine_Denmark_2015_19295_1p1_Sw-L_2015_H1N1_1A.3.3.2
MT667063_A_swine_Denmark_2014_9477_1p1_1_Sw-L_2014_H1N1_1A.3.3.2
MK367119_A_swine_Bad_Wuennenberg_20518_2014_H1N1_1A.3.3.2
MK367191_A_swine_Bad_Wuennenberg_20977_2014_H1N1_1A.3.3.2
MT666840_A_swine_Denmark_2018_15183_3_1p1_Sw-L_2018_H1N2_1A.3.3.2
MT666880_A_swine_Denmark_2018_11784_2_1p1_Sw-L_2018_H1N1_1A.3.3.2
MT666872_A_swine_Denmark_2018_11980_2_1p1_Sw-L_2018_H1N2_1A.3.3.2
MK367215_A_swine_Steinfeld_21010_2014_H1N1_1A.3.3.2
MT666999_A_swine_Denmark_2015_03655_3p1_Sw-L_2015_H1N1_1A.3.3.2
KU322847_A_swine_Italy_105389_2014_H1N1_1A.3.3.2
KR700073_A_swine_Denmark_10-2779-2_2011_H1N1_1A.3.3.2
MT667213_A_swine_Denmark_2014_10_231_1p1_Hu-L_2014_H1N1_1A.3.3.2
MT667198_A_swine_Denmark_2014_6252_2p1_Hu-L_2014_H1N2_1A.3.3.2
A_swine_Italy_19-23998-3_2019_H1N1_1A.3.3.2
A_swine_Italy_18_64849-32_2018_H1N1_1A.3.3.2
A_swine_Italy_18-109888-31_2018_H1N1_1A.3.3.2
A_swine_Italy_18-82770-28_2018_H1N1_1A.3.3.2
A_swine_Italy_361699_2019_H1N1_1A.3.3.2
A_swine_Italy_76704_2019_H1N1_1A.3.3.2
A_swine_Italy_20-17132-10_2020_H1N1_1A.3.3.2
A_swine_Italy_297520_2018_H1N1_1A.3.3.2
A_swine_Italy_18-57405-3_2018_H1N1_1A.3.3.2
KU323245_A_swine_Italy_179057_2015_H1N1_1A.3.3.2
KU322687_A_swine_Italy_282866_2013_H1N1_1A.3.3.2
KR700807_A_swine_England_060969_2013_H1N1_1A.3.3.2
MK367047_A_swine_Bothenheilingen_19699_2014_H1N1_1A.3.3.2
KR701025_A_swine_Finland_si632_2013_H1N1_1A.3.3.2
A_swine_Italy_406037_2019_H1N2_1A.3.3.2
A_swine_Italy_363738_2019_H1N1_1A.3.3.2
MT330584_A_Alaska_02_2020
MK960127_A_Italy_9368_2019
MT244105_A_Alabama_03_2020
A_swine_Italy_18-45794-37_2018_H1N1_1A.3.3.2
A_swine_Italy_60023_2019_H1N1_1A.3.3.2
A_swine_Italy_26034_2019_H1N1_1A.3.3.2
MT331578_A_Wyoming_05_2020
A_swine_Italy_81938_2019_H1N1_1A.3.3.2
MK773010_A_California_52_2019_H1N1_1A.3.3.2

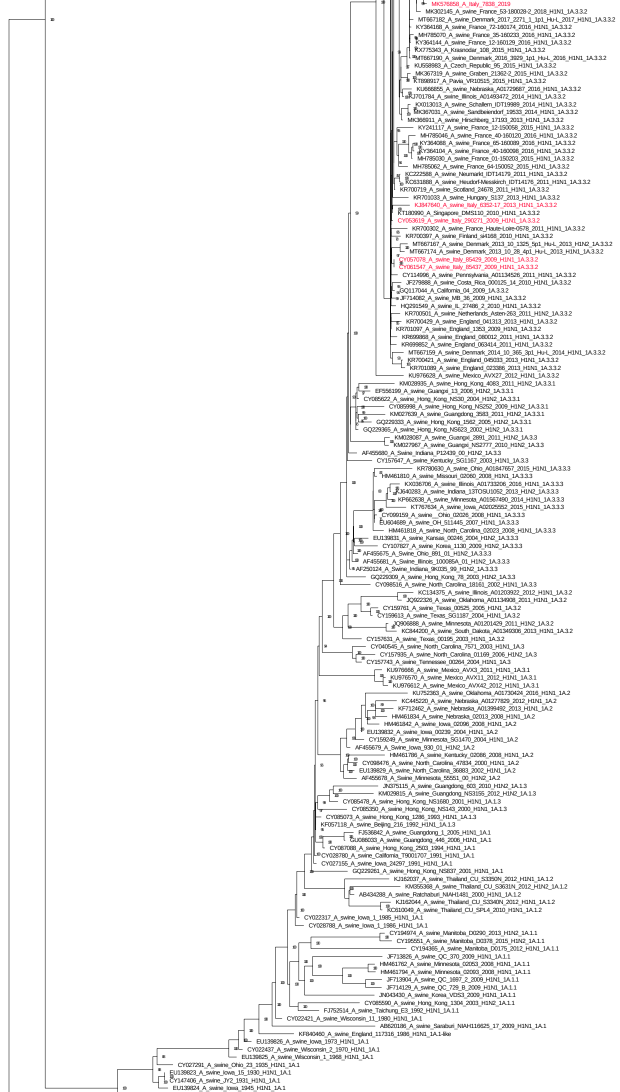
MK576858_A_Italy_7838_2019
MK302145_A_swine_France_53-180028-2_2018_H1N1_1A.3.3.2
MT667182_A_swine_Denmark_2017_2271_1_1p1_Hu-L_2017_H1N1_1A.3.3.2
KY364168_A_swine_France_72-160174_2016_H1N1_1A.3.3.2
MH785070_A_swine_France_35-160233_2016_H1N1_1A.3.3.2
KY364144_A_swine_France_12-160129_2016_H1N1_1A.3.3.2
KX775343_A_Krasnodar_108_2015_H1N1_1A.3.3.2
MT667190_A_swine_Denmark_2016_3929_1p1_Hu-L_2016_H1N1_1A.3.3.2
KU558983_A_Czech_Republic_95_2015_H1N1_1A.3.3.2
MK367319_A_swine_Graben_21362-2_2015_H1N1_1A.3.3.2
KT898917_A_Pavia_VR10515_2015_H1N1_1A.3.3.2
KU666855_A_swine_Nebraska_A01729687_2016_H1N1_1A.3.3.2
KJ701784_A_swine_Illinois_A01493472_2014_H1N1_1A.3.3.2
KX013013_A_swine_Schallern_IDT19989_2014_H1N1_1A.3.3.2
MK367031_A_swine_Sandbeiendorf_19533_2014_H1N1_1A.3.3.2
MK366911_A_swine_Hirschberg_17193_2013_H1N1_1A.3.3.2
KY241117_A_swine_France_12-150058_2015_H1N1_1A.3.3.2
MH785046_A_swine_France_40-160120_2016_H1N1_1A.3.3.2
KY364088_A_swine_France_65-160089_2016_H1N1_1A.3.3.2
KY364104_A_swine_France_40-160098_2016_H1N1_1A.3.3.2
MH785030_A_swine_France_01-150203_2015_H1N1_1A.3.3.2
MH785062_A_swine_France_64-150052_2015_H1N1_1A.3.3.2
KC222588_A_swine_Neumarkt_IDT14179_2011_H1N1_1A.3.3.2
KC631888_A_swine_Heudorf-Messkirch_IDT14176_2011_H1N1_1A.3.3.2
KR700719_A_swine_Scotland_24678_2011_H1N1_1A.3.3.2
KR701033_A_swine_Hungary_S137_2013_H1N1_1A.3.3.2
KJ847640_A_swine_Italy_6352-17_2013_H1N1_1A.3.3.2
KT180990_A_Singapore_DMS110_2010_H1N1_1A.3.3.2
CY053619_A_swine_Italy_290271_2009_H1N1_1A.3.3.2
KR700302_A_swine_France_Haute-Loire-0578_2011_H1N1_1A.3.3.2
KR700397_A_swine_Finland_si4168_2010_H1N1_1A.3.3.2
MT667167_A_swine_Denmark_2013_10_1325_5p1_Hu-L_2013_H1N2_1A.3.3.2
MT667174_A_swine_Denmark_2013_10_28_4p1_Hu-L_2013_H1N1_1A.3.3.2
CY057078_A_swine_Italy_85429_2009_H1N1_1A.3.3.2
CY061547_A_swine_Italy_85437_2009_H1N1_1A.3.3.2
CY114996_A_swine_Pennsylvania_A01134526_2011_H1N1_1A.3.3.2
JF279888_A_swine_Costa_Rica_000125_14_2010_H1N1_1A.3.3.2
GQ117044_A_California_04_2009_1A.3.3.2
JF714082_A_swine_MB_36_2009_H1N1_1A.3.3.2
HQ291549_A_swine_IL_27486_2_2010_H1N1_1A.3.3.2
KR700501_A_swine_Netherlands_Asten-263_2011_H1N2_1A.3.3.2
KR700429_A_swine_England_041313_2013_H1N1_1A.3.3.2
KR701097_A_swine_England_1353_2009_H1N1_1A.3.3.2
KR699868_A_swine_England_080012_2011_H1N1_1A.3.3.2
KR699852_A_swine_England_063414_2011_H1N1_1A.3.3.2
MT667159_A_swine_Denmark_2014_10_365_3p1_Hu-L_2014_H1N1_1A.3.3.2
KR700421_A_swine_England_045033_2013_H1N1_1A.3.3.2
KR701089_A_swine_England_023386_2013_H1N1_1A.3.3.2
KU976628_A_swine_Mexico_AVX27_2012_H1N1_1A.3.3.2
KM028935_A_swine_Hong_Kong_4083_2011_H1N2_1A.3.3.1
EF556199_A_swine_Guangxi_13_2006_H1N2_1A.3.3.1
CY085622_A_swine_Hong_Kong_NS30_2004_H1N2_1A.3.3.1
CY085998_A_swine_Hong_Kong_NS252_2009_H1N2_1A.3.3.1
KM027639_A_swine_Guangdong_3583_2011_H1N2_1A.3.3.1
GQ229333_A_swine_Hong_Kong_1562_2005_H1N2_1A.3.3.1
GQ229365_A_swine_Hong_Kong_NS623_2002_H1N2_1A.3.3.1
KM028087_A_swine_Guangxi_2891_2011_H1N2_1A.3.3
KM027967_A_swine_Guangxi_NS2777_2010_H1N2_1A.3.3
AF455680_A_Swine_Indiana_P12439_00_H1N2_1A.3.3
CY157647_A_swine_Kentucky_SG1167_2003_H1N1_1A.3.3
KR780630_A_swine_Ohio_A01847657_2015_H1N1_1A.3.3.3
HM461810_A_swine_Missouri_02060_2008_H1N1_1A.3.3.3
KX036706_A_swine_Illinois_A01733206_2016_H1N1_1A.3.3.3
KJ640283_A_swine_Indiana_13TOSU1052_2013_H1N2_1A.3.3.3
KP662638_A_swine_Minnesota_A01567490_2014_H1N1_1A.3.3.3
KT767634_A_swine_Iowa_A02025552_2015_H1N1_1A.3.3.3
CY099159_A_swine__Ohio_02026_2008_H1N1_1A.3.3.3
EU604689_A_swine_OH_511445_2007_H1N1_1A.3.3.3
HM461818_A_swine_North_Carolina_02023_2008_H1N1_1A.3.3.3
EU139831_A_swine_Kansas_00246_2004_H1N2_1A.3.3.3
CY107827_A_swine_Korea_1130_2009_H1N2_1A.3.3.3
AF455675_A_Swine_Ohio_891_01_H1N2_1A.3.3.3
AF455681_A_Swine_Illinois_100085A_01_H1N2_1A.3.3.3
AF250124_A_Swine_Indiana_9K035_99_H1N2_1A.3.3.3
GQ229309_A_swine_Hong_Kong_78_2003_H1N2_1A.3.3
CY098516_A_swine_North_Carolina_18161_2002_H1N1_1A.3.3
KC134375_A_swine_Illinois_A01203922_2012_H1N1_1A.3.2
JQ922326_A_swine_Oklahoma_A01134908_2011_H1N1_1A.3.2
CY159761_A_swine_Texas_00525_2005_H1N1_1A.3.2
CY159613_A_swine_Texas_SG1187_2004_H1N1_1A.3.2
JQ906888_A_swine_Minnesota_A01201429_2011_H1N2_1A.3.2
KC844200_A_swine_South_Dakota_A01349306_2013_H1N1_1A.3.2
CY157631_A_swine_Texas_00195_2003_H1N1_1A.3.2
CY040545_A_swine_North_Carolina_7571_2003_H1N1_1A.3
CY157935_A_swine_North_Carolina_01169_2006_H1N2_1A.3
CY157743_A_swine_Tennessee_00264_2004_H1N1_1A.3
KU976666_A_swine_Mexico_AVX3_2011_H1N1_1A.3.1
KU976570_A_swine_Mexico_AVX11_2012_H1N1_1A.3.1
KU976612_A_swine_Mexico_AVX42_2012_H1N1_1A.3.1
KU752363_A_swine_Oklahoma_A01730424_2016_H1N1_1A.2
KC445220_A_swine_Nebraska_A01277829_2012_H1N1_1A.2
KF712462_A_swine_Nebraska_A01399492_2013_H1N1_1A.2
HM461834_A_swine_Nebraska_02013_2008_H1N1_1A.2
HM461842_A_swine_Iowa_02096_2008_H1N1_1A.2
EU139832_A_swine_Iowa_00239_2004_H1N1_1A.2
CY159249_A_swine_Minnesota_SG1470_2004_H1N1_1A.2
AF455679_A_Swine_Iowa_930_01_H1N2_1A.2
HM461786_A_swine_Kentucky_02086_2008_H1N1_1A.2
CY098476_A_swine_North_Carolina_47834_2000_H1N1_1A.2
EU139829_A_swine_North_Carolina_36883_2002_H1N1_1A.2
AF455678_A_Swine_Minnesota_55551_00_H1N2_1A.2
JN375115_A_swine_Guangdong_603_2010_H1N2_1A.1.3
KM029815_A_swine_Guangdong_NS3155_2012_H1N2_1A.1.3
CY085478_A_swine_Hong_Kong_NS1680_2001_H1N1_1A.1.3
CY085350_A_swine_Hong_Kong_NS143_2000_H1N1_1A.1.3
CY085073_A_swine_Hong_Kong_1286_1993_H1N1_1A.1.3
KF057118_A_swine_Beijing_216_1992_H1N1_1A.1.3
FJ536842_A_swine_Guangdong_1_2005_H1N1_1A.1
GU086033_A_swine_Guangdong_446_2006_H1N1_1A.1
CY087088_A_swine_Hong_Kong_2503_1994_H1N1_1A.1
CY028780_A_swine_California_T9001707_1991_H1N1_1A.1
CY027155_A_swine_Iowa_24297_1991_H1N1_1A.1
GQ229261_A_swine_Hong_Kong_NS837_2001_H1N1_1A.1
KJ162037_A_swine_Thailand_CU_S3350N_2012_H1N1_1A.1.2
KM355368_A_swine_Thailand_CU_S3631N_2012_H1N2_1A.1.2
AB434288_A_swine_Ratchaburi_NIAH1481_2000_H1N1_1A.1.2
KJ162044_A_swine_Thailand_CU_S3340N_2012_H1N1_1A.1.2
KC610049_A_swine_Thailand_CU_SPL4_2010_H1N1_1A.1.2
CY022317_A_swine_Iowa_1_1985_H1N1_1A.1
CY028788_A_swine_Iowa_1_1986_H1N1_1A.1
CY194974_A_swine_Manitoba_D0290_2013_H1N2_1A.1.1
CY195551_A_swine_Manitoba_D0378_2015_H1N2_1A.1.1
CY194365_A_swine_Manitoba_D0175_2012_H1N1_1A.1.1
JF713826_A_swine_QC_370_2009_H1N1_1A.1.1
HM461762_A_swine_Minnesota_02053_2008_H1N1_1A.1.1
HM461794_A_swine_Minnesota_02093_2008_H1N1_1A.1.1
JF713904_A_swine_QC_1697_2_2009_H1N1_1A.1.1
JF714129_A_swine_QC_729_B_2009_H1N1_1A.1.1
JN043430_A_swine_Korea_VDS3_2009_H1N1_1A.1.1
CY085590_A_swine_Hong_Kong_1304_2003_H1N2_1A.1.1
FJ752514_A_swine_Taichung_E3_1992_H1N1_1A.1.1
CY022421_A_swine_Wisconsin_11_1980_H1N1_1A.1
AB620186_A_swine_Saraburi_NIAH116625_17_2009_H1N1_1A.1
KF840460_A_swine_England_117316_1986_H1N1_1A.1-like
EU139826_A_swine_Iowa_1973_H1N1_1A.1
CY022437_A_swine_Wisconsin_2_1970_H1N1_1A.1
EU139825_A_swine_Wisconsin_1_1968_H1N1_1A.1
CY027291_A_swine_Ohio_23_1935_H1N1_1A.1
EU139823_A_swine_Iowa_15_1930_H1N1_1A.1
CY147406_A_swine_JY2_1931_H1N1_1A.1
EU139824_A_swine_Iowa_1945_H1N1_1A.1

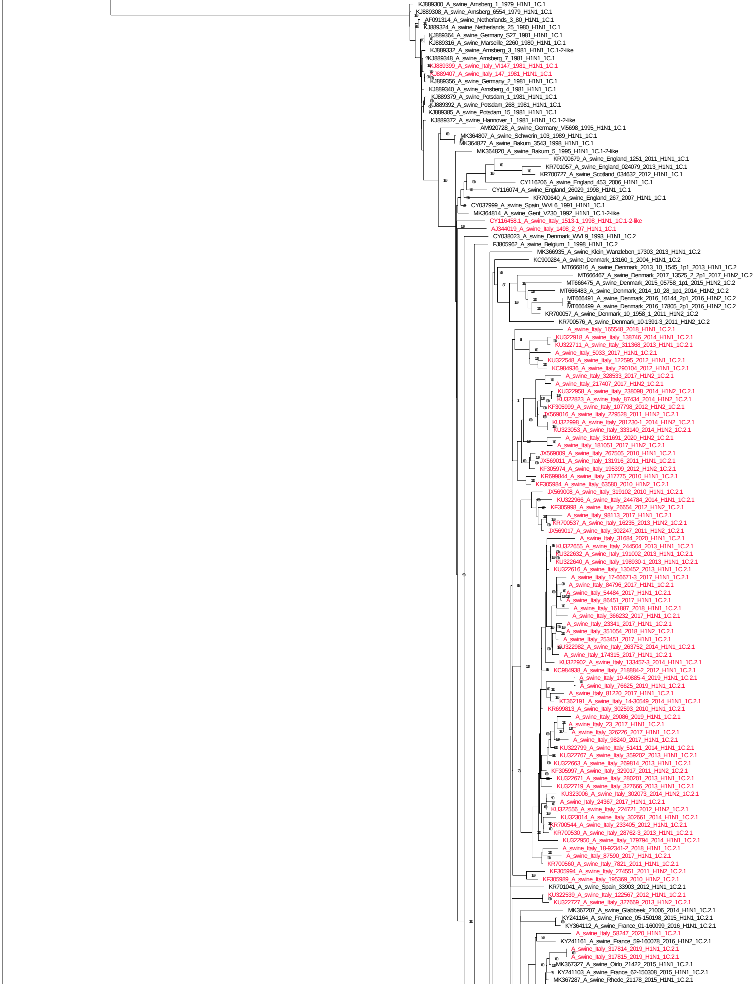

KJ889300_A_swine_Arnsberg_1_1979_H1N1_1C.1
KJ889308_A_swine_Arnsberg_6554_1979_H1N1_1C.1
AF091314_A_swine_Netherlands_3_80_H1N1_1C.1
KJ889324_A_swine_Netherlands_25_1980_H1N1_1C.1
KJ889364_A_swine_Germany_S27_1981_H1N1_1C.1
KJ889316_A_swine_Marseille_2260_1980_H1N1_1C.1
KJ889332_A_swine_Arnsberg_3_1981_H1N1_1C.1-2-like
KJ889348_A_swine_Arnsberg_7_1981_H1N1_1C.1
KJ889399_A_swine_Italy_VI147_1981_H1N1_1C.1
KJ889407_A_swine_Italy_147_1981_H1N1_1C.1
KJ889356_A_swine_Germany_2_1981_H1N1_1C.1
KJ889340_A_swine_Arnsberg_4_1981_H1N1_1C.1
KJ889379_A_swine_Potsdam_1_1981_H1N1_1C.1
KJ889392_A_swine_Potsdam_268_1981_H1N1_1C.1
KJ889385_A_swine_Potsdam_15_1981_H1N1_1C.1
KJ889372_A_swine_Hannover_1_1981_H1N1_1C.1-2-like
AM920728_A_swine_Germany_Vi5698_1995_H1N1_1C.1
MK364807_A_swine_Schwerin_103_1989_H1N1_1C.1
MK364827_A_swine_Bakum_3543_1998_H1N1_1C.1
MK364820_A_swine_Bakum_5_1995_H1N1_1C.1-2-like
KR700679_A_swine_England_1251_2011_H1N1_1C.1
KR701057_A_swine_England_024079_2013_H1N1_1C.1
KR700727_A_swine_Scotland_034632_2012_H1N1_1C.1
CY116206_A_swine_England_453_2006_H1N1_1C.1
CY116074_A_swine_England_26029_1998_H1N1_1C.1
KR700640_A_swine_England_267_2007_H1N1_1C.1
CY037999_A_swine_Spain_WVL6_1991_H1N1_1C.1
MK364814_A_swine_Gent_V230_1992_H1N1_1C.1-2-like
CY116458.1_A_swine_Italy_1513-1_1998_H1N1_1C.1-2-like
AJ344019_A_swine_Italy_1498_2_97_H1N1_1C.1
CY038023_A_swine_Denmark_WVL9_1993_H1N1_1C.2
FJ805962_A_swine_Belgium_1_1998_H1N1_1C.2
MK366935_A_swine_Klein_Wanzleben_17303_2013_H1N1_1C.2
KC900284_A_swine_Denmark_13160_1_2004_H1N1_1C.2
MT666816_A_swine_Denmark_2013_10_1545_1p1_2013_H1N1_1C.2
MT666467_A_swine_Denmark_2017_13525_2_2p1_2017_H1N2_1C.2
MT666475_A_swine_Denmark_2015_05758_1p1_2015_H1N2_1C.2
MT666483_A_swine_Denmark_2014_10_28_1p1_2014_H1N2_1C.2
MT666491_A_swine_Denmark_2016_16144_2p1_2016_H1N2_1C.2
MT666499_A_swine_Denmark_2016_17805_2p1_2016_H1N2_1C.2
KR700057_A_swine_Denmark_10_1958_1_2011_H1N2_1C.2
KR700576_A_swine_Denmark_10-1391-3_2011_H1N2_1C.2
A_swine_Italy_165548_2018_H1N1_1C.2.1
KU322918_A_swine_Italy_138746_2014_H1N1_1C.2.1
KU322711_A_swine_Italy_311368_2013_H1N1_1C.2.1
A_swine_Italy_5033_2017_H1N1_1C.2.1
KU322548_A_swine_Italy_122595_2012_H1N1_1C.2.1
KC984936_A_swine_Italy_290104_2012_H1N1_1C.2.1
A_swine_Italy_328533_2017_H1N2_1C.2.1
A_swine_Italy_217407_2017_H1N2_1C.2.1
KU322958_A_swine_Italy_238098_2014_H1N2_1C.2.1
KU322823_A_swine_Italy_87434_2014_H1N2_1C.2.1
KF305999_A_swine_Italy_107798_2012_H1N2_1C.2.1
JX569016_A_swine_Italy_229528_2011_H1N2_1C.2.1
KU322998_A_swine_Italy_281230-1_2014_H1N2_1C.2.1
KU323053_A_swine_Italy_333140_2014_H1N2_1C.2.1
A_swine_Italy_311691_2020_H1N2_1C.2.1
A_swine_Italy_181051_2017_H1N2_1C.2.1
JX569009_A_swine_Italy_267505_2010_H1N1_1C.2.1
JX569011_A_swine_Italy_131916_2011_H1N1_1C.2.1
KF305974_A_swine_Italy_195399_2012_H1N2_1C.2.1
KR699844_A_swine_Italy_317775_2010_H1N1_1C.2.1
KF305984_A_swine_Italy_63580_2010_H1N2_1C.2.1
JX569008_A_swine_Italy_319102_2010_H1N1_1C.2.1
KU322966_A_swine_Italy_244784_2014_H1N1_1C.2.1
KF305998_A_swine_Italy_26654_2012_H1N2_1C.2.1
A_swine_Italy_98113_2017_H1N1_1C.2.1
KR700537_A_swine_Italy_16235_2013_H1N2_1C.2.1
JX569017_A_swine_Italy_302247_2011_H1N2_1C.2.1
A_swine_Italy_31684_2020_H1N1_1C.2.1
KU322655_A_swine_Italy_244504_2013_H1N1_1C.2.1
KU322632_A_swine_Italy_191002_2013_H1N1_1C.2.1
KU322640_A_swine_Italy_198930-1_2013_H1N1_1C.2.1
KU322616_A_swine_Italy_130452_2013_H1N1_1C.2.1
A_swine_Italy_17-66671-3_2017_H1N1_1C.2.1
A_swine_Italy_84796_2017_H1N1_1C.2.1
A_swine_Italy_54484_2017_H1N1_1C.2.1
A_swine_Italy_86451_2017_H1N1_1C.2.1
A_swine_Italy_161887_2018_H1N1_1C.2.1
A_swine_Italy_366232_2017_H1N1_1C.2.1
A_swine_Italy_23341_2017_H1N1_1C.2.1
A_swine_Italy_351054_2018_H1N2_1C.2.1
A_swine_Italy_253451_2017_H1N1_1C.2.1
KU322982_A_swine_Italy_263752_2014_H1N1_1C.2.1
A_swine_Italy_174315_2017_H1N1_1C.2.1
KU322902_A_swine_Italy_133457-3_2014_H1N1_1C.2.1
KC984938_A_swine_Italy_218884-2_2012_H1N1_1C.2.1
A_swine_Italy_19-49885-4_2019_H1N1_1C.2.1
A_swine_Italy_76625_2019_H1N1_1C.2.1
A_swine_Italy_81220_2017_H1N1_1C.2.1
KT362191_A_swine_Italy_14-30549_2014_H1N1_1C.2.1
KR699813_A_swine_Italy_302593_2010_H1N1_1C.2.1
A_swine_Italy_29086_2019_H1N1_1C.2.1
A_swine_Italy_23_2017_H1N1_1C.2.1
A_swine_Italy_326226_2017_H1N1_1C.2.1
A_swine_Italy_98240_2017_H1N1_1C.2.1
KU322799_A_swine_Italy_51411_2014_H1N1_1C.2.1
KU322767_A_swine_Italy_359202_2013_H1N1_1C.2.1
KU322663_A_swine_Italy_269814_2013_H1N1_1C.2.1
KF305997_A_swine_Italy_329017_2011_H1N2_1C.2.1
KU322671_A_swine_Italy_280201_2013_H1N1_1C.2.1
KU322719_A_swine_Italy_327666_2013_H1N1_1C.2.1
KU323006_A_swine_Italy_302073_2014_H1N2_1C.2.1
A_swine_Italy_24367_2017_H1N1_1C.2.1
KU322556_A_swine_Italy_224721_2012_H1N2_1C.2.1
KU323014_A_swine_Italy_302661_2014_H1N1_1C.2.1
KR700544_A_swine_Italy_233405_2012_H1N1_1C.2.1
KR700530_A_swine_Italy_28762-3_2013_H1N1_1C.2.1
KU322950_A_swine_Italy_179794_2014_H1N1_1C.2.1
A_swine_Italy_18-92341-2_2018_H1N1_1C.2.1
A_swine_Italy_87590_2017_H1N1_1C.2.1
KR700560_A_swine_Italy_7821_2011_H1N1_1C.2.1
KF305994_A_swine_Italy_274551_2011_H1N2_1C.2.1
KF305989_A_swine_Italy_195369_2010_H1N2_1C.2.1
KR701041_A_swine_Spain_33903_2012_H1N1_1C.2.1
KU322539_A_swine_Italy_122567_2012_H1N1_1C.2.1
KU322727_A_swine_Italy_327669_2013_H1N2_1C.2.1
MK367207_A_swine_Glabbeek_21006_2014_H1N1_1C.2.1
KY241164_A_swine_France_05-150198_2015_H1N1_1C.2.1
KY364112_A_swine_France_01-160099_2016_H1N1_1C.2.1
A_swine_Italy_58247_2020_H1N1_1C.2.1
KY241161_A_swine_France_59-160078_2016_H1N2_1C.2.1
A_swine_Italy_317814_2019_H1N1_1C.2.1
A_swine_Italy_317815_2019_H1N1_1C.2.1
MK367327_A_swine_Oirlo_21422_2015_H1N1_1C.2.1
KY241103_A_swine_France_62-150308_2015_H1N1_1C.2.1
MK367287_A_swine_Rhede_21178_2015_H1N1_1C.2.1

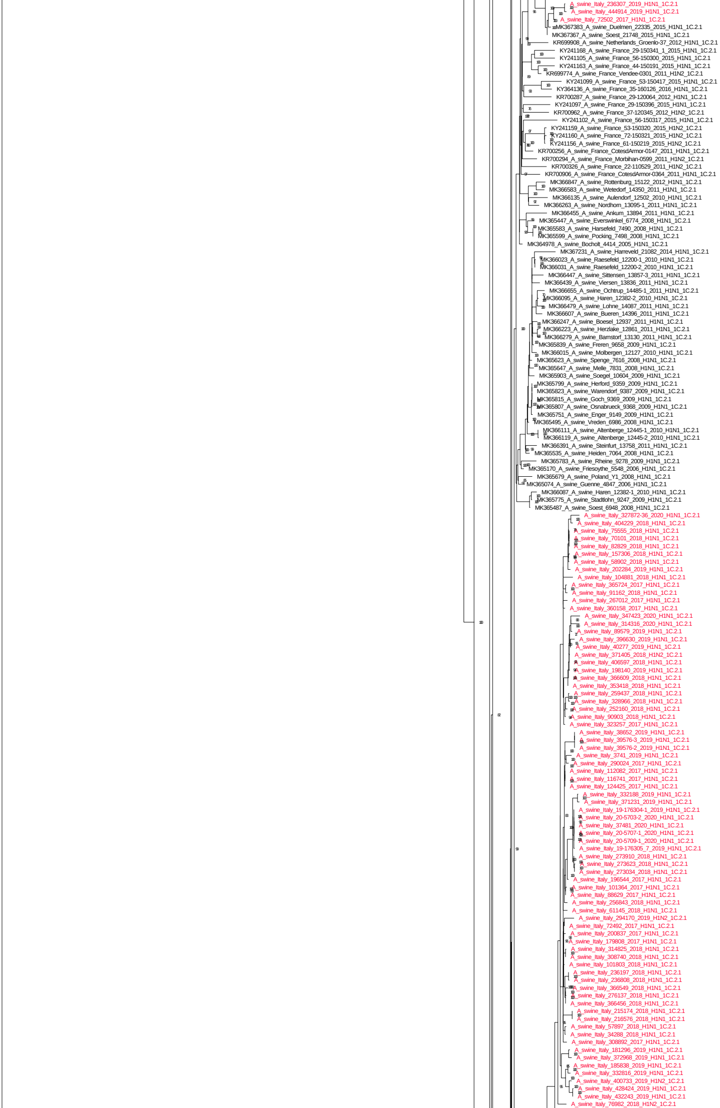
A_swine_Italy_236307_2019_H1N1_1C.2.1
A_swine_Italy_444914_2019_H1N1_1C.2.1
A_swine_Italy_72502_2017_H1N1_1C.2.1
MK367383_A_swine_Duelmen_22335_2015_H1N1_1C.2.1
MK367367_A_swine_Soest_21748_2015_H1N1_1C.2.1
KR699908_A_swine_Netherlands_Groenlo-37_2012_H1N1_1C.2.1
KY241168_A_swine_France_29-150341_1_2015_H1N1_1C.2.1
KY241105_A_swine_France_56-150300_2015_H1N1_1C.2.1
KY241163_A_swine_France_44-150191_2015_H1N1_1C.2.1
KR699774_A_swine_France_Vendee-0301_2011_H1N2_1C.2.1
KY241099_A_swine_France_53-150417_2015_H1N1_1C.2.1
KY364136_A_swine_France_35-160126_2016_H1N1_1C.2.1
KR700287_A_swine_France_29-120064_2012_H1N1_1C.2.1
KY241097_A_swine_France_29-150396_2015_H1N1_1C.2.1
KR700962_A_swine_France_37-120345_2012_H1N2_1C.2.1
KY241102_A_swine_France_56-150317_2015_H1N1_1C.2.1
KY241159_A_swine_France_53-150320_2015_H1N2_1C.2.1
KY241160_A_swine_France_72-150321_2015_H1N2_1C.2.1
KY241156_A_swine_France_61-150219_2015_H1N2_1C.2.1
KR700256_A_swine_France_CotesdArmor-0147_2011_H1N1_1C.2.1
KR700294_A_swine_France_Morbihan-0599_2011_H1N2_1C.2.1
KR700326_A_swine_France_22-110529_2011_H1N2_1C.2.1
KR700906_A_swine_France_CotesdArmor-0364_2011_H1N1_1C.2.1
MK366847_A_swine_Rottenburg_15122_2012_H1N1_1C.2.1
MK366583_A_swine_Wetedorf_14350_2011_H1N1_1C.2.1
MK366135_A_swine_Aulendorf_12502_2010_H1N1_1C.2.1
MK366263_A_swine_Nordhorn_13095-1_2011_H1N1_1C.2.1
MK366455_A_swine_Ankum_13894_2011_H1N1_1C.2.1
MK365447_A_swine_Everswinkel_6774_2008_H1N1_1C.2.1
MK365583_A_swine_Harsefeld_7490_2008_H1N1_1C.2.1
MK365599_A_swine_Pocking_7498_2008_H1N1_1C.2.1
MK364978_A_swine_Bocholt_4414_2005_H1N1_1C.2.1
MK367231_A_swine_Harreveld_21082_2014_H1N1_1C.2.1
MK366023_A_swine_Raesefeld_12200-1_2010_H1N1_1C.2.1
MK366031_A_swine_Raesefeld_12200-2_2010_H1N1_1C.2.1
MK366447_A_swine_Sittensen_13857-3_2011_H1N1_1C.2.1
MK366439_A_swine_Viersen_13836_2011_H1N1_1C.2.1
MK366655_A_swine_Ochtrup_14485-1_2011_H1N1_1C.2.1
MK366095_A_swine_Haren_12382-2_2010_H1N1_1C.2.1
MK366479_A_swine_Lohne_14087_2011_H1N1_1C.2.1
MK366607_A_swine_Bueren_14396_2011_H1N1_1C.2.1
MK366247_A_swine_Boesel_12937_2011_H1N1_1C.2.1
MK366223_A_swine_Herzlake_12861_2011_H1N1_1C.2.1
MK366279_A_swine_Barnstorf_13130_2011_H1N1_1C.2.1
MK365839_A_swine_Freren_9658_2009_H1N1_1C.2.1
MK366015_A_swine_Molbergen_12127_2010_H1N1_1C.2.1
MK365623_A_swine_Spenge_7616_2008_H1N1_1C.2.1
MK365647_A_swine_Melle_7831_2008_H1N1_1C.2.1
MK365903_A_swine_Soegel_10604_2009_H1N1_1C.2.1
MK365799_A_swine_Herford_9359_2009_H1N1_1C.2.1
MK365823_A_swine_Warendorf_9387_2009_H1N1_1C.2.1
MK365815_A_swine_Goch_9369_2009_H1N1_1C.2.1
MK365807_A_swine_Osnabrueck_9368_2009_H1N1_1C.2.1
MK365751_A_swine_Enger_9149_2009_H1N1_1C.2.1
MK365495_A_swine_Vreden_6986_2008_H1N1_1C.2.1
MK366111_A_swine_Altenberge_12445-1_2010_H1N1_1C.2.1
MK366119_A_swine_Altenberge_12445-2_2010_H1N1_1C.2.1
MK366391_A_swine_Steinfurt_13758_2011_H1N1_1C.2.1
MK365535_A_swine_Heiden_7064_2008_H1N1_1C.2.1
MK365783_A_swine_Rheine_9278_2009_H1N1_1C.2.1
MK365170_A_swine_Friesoythe_5548_2006_H1N1_1C.2.1
MK365679_A_swine_Poland_Y1_2008_H1N1_1C.2.1
MK365074_A_swine_Guenne_4847_2006_H1N1_1C.2.1
MK366087_A_swine_Haren_12382-1_2010_H1N1_1C.2.1
MK365775_A_swine_Stadtlohn_9247_2009_H1N1_1C.2.1
MK365487_A_swine_Soest_6948_2008_H1N1_1C.2.1
A_swine_Italy_327872-36_2020_H1N1_1C.2.1
A_swine_Italy_404229_2018_H1N1_1C.2.1
A_swine_Italy_75555_2018_H1N1_1C.2.1
A_swine_Italy_70101_2018_H1N1_1C.2.1
A_swine_Italy_82829_2018_H1N1_1C.2.1
A_swine_Italy_157306_2018_H1N1_1C.2.1
A_swine_Italy_58902_2018_H1N1_1C.2.1
A_swine_Italy_202284_2019_H1N1_1C.2.1
A_swine_Italy_104881_2018_H1N1_1C.2.1
A_swine_Italy_365724_2017_H1N1_1C.2.1
A_swine_Italy_91162_2018_H1N1_1C.2.1
A_swine_Italy_267012_2017_H1N1_1C.2.1
A_swine_Italy_360158_2017_H1N1_1C.2.1
A_swine_Italy_347423_2020_H1N1_1C.2.1
A_swine_Italy_314316_2020_H1N1_1C.2.1
A_swine_Italy_89579_2019_H1N1_1C.2.1
A_swine_Italy_396630_2019_H1N1_1C.2.1
A_swine_Italy_40277_2019_H1N1_1C.2.1
A_swine_Italy_371405_2018_H1N2_1C.2.1
A_swine_Italy_406597_2018_H1N1_1C.2.1
A_swine_Italy_198140_2019_H1N1_1C.2.1
A_swine_Italy_366609_2018_H1N1_1C.2.1
A_swine_Italy_353418_2018_H1N1_1C.2.1
A_swine_Italy_259437_2018_H1N1_1C.2.1
A_swine_Italy_328966_2018_H1N1_1C.2.1
A_swine_Italy_252160_2018_H1N1_1C.2.1
A_swine_Italy_90903_2018_H1N1_1C.2.1
A_swine_Italy_323257_2017_H1N1_1C.2.1
A_swine_Italy_38652_2019_H1N1_1C.2.1
A_swine_Italy_39576-3_2019_H1N1_1C.2.1
A_swine_Italy_39576-2_2019_H1N1_1C.2.1
A_swine_Italy_3741_2019_H1N1_1C.2.1
A_swine_Italy_290024_2017_H1N1_1C.2.1
A_swine_Italy_112082_2017_H1N1_1C.2.1
A_swine_Italy_116741_2017_H1N1_1C.2.1
A_swine_Italy_124425_2017_H1N1_1C.2.1
A_swine_Italy_332188_2019_H1N1_1C.2.1
A_swine_Italy_371231_2019_H1N1_1C.2.1
A_swine_Italy_19-176304-1_2019_H1N1_1C.2.1
A_swine_Italy_20-5703-2_2020_H1N1_1C.2.1
A_swine_Italy_37481_2020_H1N1_1C.2.1
A_swine_Italy_20-5707-1_2020_H1N1_1C.2.1
A_swine_Italy_20-5709-1_2020_H1N1_1C.2.1
A_swine_Italy_19-176305_7_2019_H1N1_1C.2.1
A_swine_Italy_273910_2018_H1N1_1C.2.1
A_swine_Italy_273623_2018_H1N1_1C.2.1
A_swine_Italy_273034_2018_H1N1_1C.2.1
A_swine_Italy_196544_2017_H1N1_1C.2.1
A_swine_Italy_101364_2017_H1N1_1C.2.1
A_swine_Italy_88629_2017_H1N1_1C.2.1
A_swine_Italy_256843_2018_H1N1_1C.2.1
A_swine_Italy_61145_2018_H1N1_1C.2.1
A_swine_Italy_294170_2019_H1N2_1C.2.1
A_swine_Italy_72492_2017_H1N1_1C.2.1
A_swine_Italy_200837_2017_H1N1_1C.2.1
A_swine_Italy_179808_2017_H1N1_1C.2.1
A_swine_Italy_314825_2018_H1N1_1C.2.1
A_swine_Italy_308740_2018_H1N1_1C.2.1
A_swine_Italy_101803_2018_H1N1_1C.2.1
A_swine_Italy_236197_2018_H1N1_1C.2.1
A_swine_Italy_236808_2018_H1N1_1C.2.1
A_swine_Italy_366549_2018_H1N1_1C.2.1
A_swine_Italy_276137_2018_H1N1_1C.2.1
A_swine_Italy_366456_2018_H1N1_1C.2.1
A_swine_Italy_215174_2018_H1N1_1C.2.1
A_swine_Italy_216576_2018_H1N1_1C.2.1
A_swine_Italy_57897_2018_H1N1_1C.2.1
A_swine_Italy_34288_2018_H1N1_1C.2.1
A_swine_Italy_308892_2017_H1N1_1C.2.1
A_swine_Italy_181296_2019_H1N1_1C.2.1
A_swine_Italy_372968_2019_H1N1_1C.2.1
A_swine_Italy_185838_2019_H1N1_1C.2.1
A_swine_Italy_332816_2019_H1N1_1C.2.1
A_swine_Italy_400733_2019_H1N2_1C.2.1
A_swine_Italy_428424_2019_H1N1_1C.2.1
A_swine_Italy_432243_2019_H1N1_1C.2.1
A_swine_Italy_76982_2018_H1N2_1C.2.1

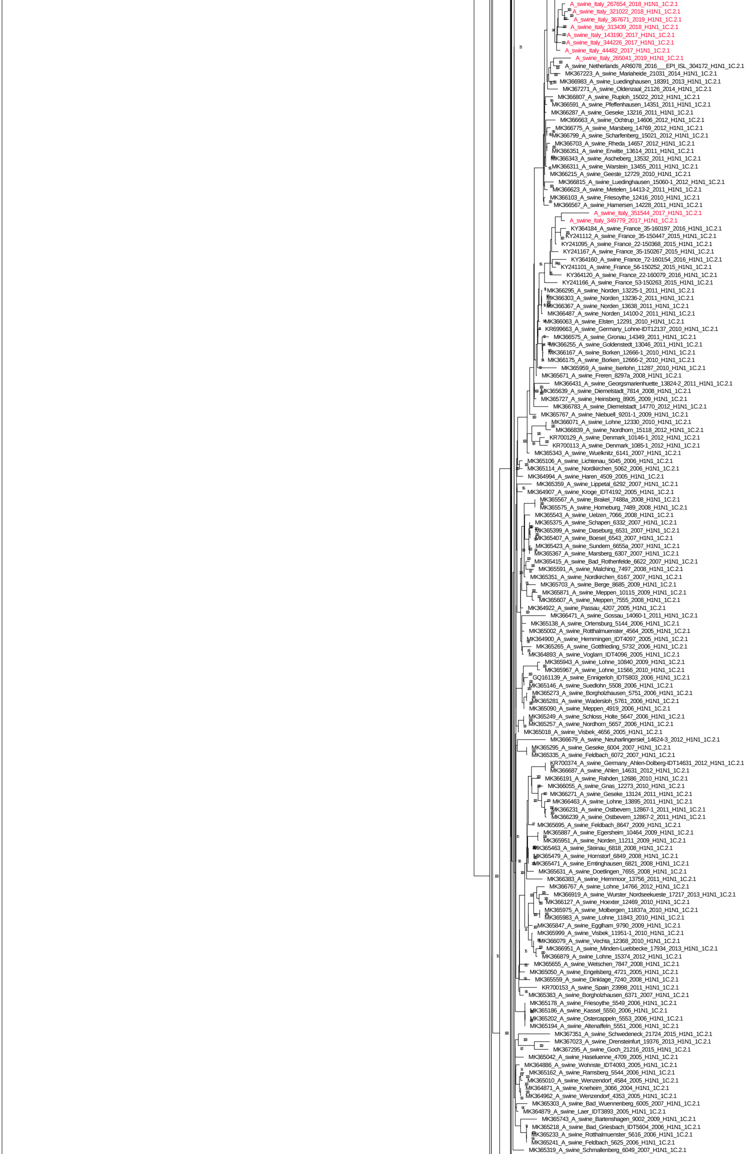

A_swine_Italy_267654_2018_H1N1_1C.2.1
A_swine_Italy_321022_2018_H1N1_1C.2.1
A_swine_Italy_367671_2019_H1N1_1C.2.1
A_swine_Italy_313439_2018_H1N1_1C.2.1
A_swine_Italy_143190_2017_H1N1_1C.2.1
A_swine_Italy_344226_2017_H1N1_1C.2.1
A_swine_Italy_44482_2017_H1N1_1C.2.1
A_swine_Italy_265041_2019_H1N1_1C.2.1
A_swine_Netherlands_AR6078_2016__EPI_ISL_304172_H1N1_1C.2.1
MK367223_A_swine_Mariaheide_21031_2014_H1N1_1C.2.1
MK366983_A_swine_Luedinghausen_18391_2013_H1N1_1C.2.1
MK367271_A_swine_Oldenzaal_21126_2014_H1N1_1C.2.1
MK366807_A_swine_Ruploh_15022_2012_H1N1_1C.2.1
MK366591_A_swine_Pfeffenhausen_14351_2011_H1N1_1C.2.1
MK366287_A_swine_Geseke_13216_2011_H1N1_1C.2.1
MK366663_A_swine_Ochtrup_14606_2012_H1N1_1C.2.1
MK366775_A_swine_Marsberg_14769_2012_H1N1_1C.2.1
MK366799_A_swine_Scharfenberg_15021_2012_H1N1_1C.2.1
MK366703_A_swine_Rheda_14657_2012_H1N1_1C.2.1
MK366351_A_swine_Erwitte_13614_2011_H1N1_1C.2.1
MK366343_A_swine_Ascheberg_13532_2011_H1N1_1C.2.1
MK366311_A_swine_Warstein_13455_2011_H1N1_1C.2.1
MK366215_A_swine_Geeste_12729_2010_H1N1_1C.2.1
MK366815_A_swine_Luedinghausen_15060-1_2012_H1N1_1C.2.1
MK366623_A_swine_Metelen_14413-2_2011_H1N1_1C.2.1
MK366103_A_swine_Friesoythe_12416_2010_H1N1_1C.2.1
MK366567_A_swine_Hamersen_14228_2011_H1N1_1C.2.1
A_swine_Italy_351544_2017_H1N1_1C.2.1
A_swine_Italy_349779_2017_H1N1_1C.2.1
KY364184_A_swine_France_35-160197_2016_H1N1_1C.2.1
KY241112_A_swine_France_35-150447_2015_H1N1_1C.2.1
KY241095_A_swine_France_22-150368_2015_H1N1_1C.2.1
KY241167_A_swine_France_35-150267_2015_H1N1_1C.2.1
KY364160_A_swine_France_72-160154_2016_H1N1_1C.2.1
KY241101_A_swine_France_56-150252_2015_H1N1_1C.2.1
KY364120_A_swine_France_22-160079_2016_H1N1_1C.2.1
KY241166_A_swine_France_53-150263_2015_H1N1_1C.2.1
MK366295_A_swine_Norden_13225-1_2011_H1N1_1C.2.1
MK366303_A_swine_Norden_13236-2_2011_H1N1_1C.2.1
MK366367_A_swine_Norden_13638_2011_H1N1_1C.2.1
MK366487_A_swine_Norden_14100-2_2011_H1N1_1C.2.1
MK366063_A_swine_Elsten_12291_2010_H1N1_1C.2.1
KR699663_A_swine_Germany_Lohne-IDT12137_2010_H1N1_1C.2.1
MK366575_A_swine_Gronau_14349_2011_H1N1_1C.2.1
MK366255_A_swine_Goldenstedt_13046_2011_H1N1_1C.2.1
MK366167_A_swine_Borken_12666-1_2010_H1N1_1C.2.1
MK366175_A_swine_Borken_12666-2_2010_H1N1_1C.2.1
MK365959_A_swine_Iserlohn_11287_2010_H1N1_1C.2.1
MK365671_A_swine_Freren_8297a_2008_H1N1_1C.2.1
MK366431_A_swine_Georgsmarienhuette_13824-2_2011_H1N1_1C.2.1
MK365639_A_swine_Diemelstadt_7814_2008_H1N1_1C.2.1
MK365727_A_swine_Heinsberg_8905_2009_H1N1_1C.2.1
MK366783_A_swine_Diemelstadt_14770_2012_H1N1_1C.2.1
MK365767_A_swine_Niebuell_9201-1_2009_H1N1_1C.2.1
MK366071_A_swine_Lohne_12330_2010_H1N1_1C.2.1
MK366839_A_swine_Nordhorn_15118_2012_H1N1_1C.2.1
KR700129_A_swine_Denmark_10146-1_2012_H1N1_1C.2.1
KR700113_A_swine_Denmark_1085-1_2012_H1N1_1C.2.1
MK365343_A_swine_Wuelknitz_6141_2007_H1N1_1C.2.1
MK365106_A_swine_Lichtenau_5045_2006_H1N1_1C.2.1
MK365114_A_swine_Nordkirchen_5062_2006_H1N1_1C.2.1
MK364994_A_swine_Haren_4509_2005_H1N1_1C.2.1
MK365359_A_swine_Lippetal_6292_2007_H1N1_1C.2.1
MK364907_A_swine_Kroge_IDT4192_2005_H1N1_1C.2.1
MK365567_A_swine_Brakel_7488a_2008_H1N1_1C.2.1
MK365575_A_swine_Horneburg_7489_2008_H1N1_1C.2.1
MK365543_A_swine_Uelzen_7066_2008_H1N1_1C.2.1
MK365375_A_swine_Schapen_6332_2007_H1N1_1C.2.1
MK365399_A_swine_Daseburg_6531_2007_H1N1_1C.2.1
MK365407_A_swine_Boesel_6543_2007_H1N1_1C.2.1
MK365423_A_swine_Sundern_6655a_2007_H1N1_1C.2.1
MK365367_A_swine_Marsberg_6307_2007_H1N1_1C.2.1
MK365415_A_swine_Bad_Rothenfelde_6622_2007_H1N1_1C.2.1
MK365591_A_swine_Malching_7497_2008_H1N1_1C.2.1
MK365351_A_swine_Nordkirchen_6167_2007_H1N1_1C.2.1
MK365703_A_swine_Berge_8685_2009_H1N1_1C.2.1
MK365871_A_swine_Meppen_10115_2009_H1N1_1C.2.1
MK365607_A_swine_Meppen_7555_2008_H1N1_1C.2.1
MK364922_A_swine_Passau_4207_2005_H1N1_1C.2.1
MK366471_A_swine_Gossau_14060-1_2011_H1N1_1C.2.1
MK365138_A_swine_Ortensburg_5144_2006_H1N1_1C.2.1
MK365002_A_swine_Rotthalmuenster_4564_2005_H1N1_1C.2.1
MK364900_A_swine_Hemmingen_IDT4097_2005_H1N1_1C.2.1
MK365265_A_swine_Gottfrieding_5732_2006_H1N1_1C.2.1
MK364893_A_swine_Voglarn_IDT4096_2005_H1N1_1C.2.1
MK365943_A_swine_Lohne_10840_2009_H1N1_1C.2.1
MK365967_A_swine_Lohne_11566_2010_H1N1_1C.2.1
GQ161139_A_swine_Ennigerloh_IDT5803_2006_H1N1_1C.2.1
MK365146_A_swine_Suedlohn_5508_2006_H1N1_1C.2.1
MK365273_A_swine_Borgholzhausen_5751_2006_H1N1_1C.2.1
MK365281_A_swine_Wadersloh_5761_2006_H1N1_1C.2.1
MK365090_A_swine_Meppen_4919_2006_H1N1_1C.2.1
MK365249_A_swine_Schloss_Holte_5647_2006_H1N1_1C.2.1
MK365257_A_swine_Nordhorn_5657_2006_H1N1_1C.2.1
MK365018_A_swine_Visbek_4656_2005_H1N1_1C.2.1
MK366679_A_swine_Neuharlingersiel_14624-3_2012_H1N1_1C.2.1
MK365295_A_swine_Geseke_6004_2007_H1N1_1C.2.1
MK365335_A_swine_Feldbach_6072_2007_H1N1_1C.2.1
KR700374_A_swine_Germany_Ahlen-Dolberg-IDT14631_2012_H1N1_1C.2.1
MK366687_A_swine_Ahlen_14631_2012_H1N1_1C.2.1
MK366191_A_swine_Rahden_12686_2010_H1N1_1C.2.1
MK366055_A_swine_Gnas_12273_2010_H1N1_1C.2.1
MK366271_A_swine_Geseke_13124_2011_H1N1_1C.2.1
MK366463_A_swine_Lohne_13895_2011_H1N1_1C.2.1
MK366231_A_swine_Ostbevern_12867-1_2011_H1N1_1C.2.1
MK366239_A_swine_Ostbevern_12867-2_2011_H1N1_1C.2.1
MK365695_A_swine_Feldbach_8647_2009_H1N1_1C.2.1
MK365887_A_swine_Egersheim_10464_2009_H1N1_1C.2.1
MK365951_A_swine_Norden_11211_2009_H1N1_1C.2.1
MK365463_A_swine_Steinau_6818_2008_H1N1_1C.2.1
MK365479_A_swine_Hornstorf_6849_2008_H1N1_1C.2.1
MK365471_A_swine_Emtinghausen_6821_2008_H1N1_1C.2.1
MK365631_A_swine_Doetlingen_7655_2008_H1N1_1C.2.1
MK366383_A_swine_Hemmoor_13756_2011_H1N1_1C.2.1
MK366767_A_swine_Lohne_14766_2012_H1N1_1C.2.1
MK366919_A_swine_Wurster_Nordseekueste_17217_2013_H1N1_1C.2.1
MK366127_A_swine_Hoexter_12469_2010_H1N1_1C.2.1
MK365975_A_swine_Molbergen_11837a_2010_H1N1_1C.2.1
MK365983_A_swine_Lohne_11843_2010_H1N1_1C.2.1
MK365847_A_swine_Egglham_9790_2009_H1N1_1C.2.1
MK365999_A_swine_Visbek_11951-1_2010_H1N1_1C.2.1
MK366079_A_swine_Vechta_12368_2010_H1N1_1C.2.1
MK366951_A_swine_Minden-Luebbecke_17934_2013_H1N1_1C.2.1
MK366879_A_swine_Lohne_15374_2012_H1N1_1C.2.1
MK365655_A_swine_Wetschen_7847_2008_H1N1_1C.2.1
MK365050_A_swine_Engelsberg_4721_2005_H1N1_1C.2.1
MK365559_A_swine_Dinklage_7240_2008_H1N1_1C.2.1
KR700153_A_swine_Spain_23998_2011_H1N1_1C.2.1
MK365383_A_swine_Borgholzhausen_6371_2007_H1N1_1C.2.1
MK365178_A_swine_Friesoythe_5549_2006_H1N1_1C.2.1
MK365186_A_swine_Kassel_5550_2006_H1N1_1C.2.1
MK365202_A_swine_Ostercappeln_5553_2006_H1N1_1C.2.1
MK365194_A_swine_Altenaffeln_5551_2006_H1N1_1C.2.1
MK367351_A_swine_Schwedeneck_21724_2015_H1N1_1C.2.1
MK367023_A_swine_Drensteinfurt_19376_2013_H1N1_1C.2.1
MK367295_A_swine_Goch_21216_2015_H1N1_1C.2.1
MK365042_A_swine_Haseluenne_4709_2005_H1N1_1C.2.1
MK364886_A_swine_Wohnste_IDT4093_2005_H1N1_1C.2.1
MK365162_A_swine_Ramsberg_5544_2006_H1N1_1C.2.1
MK365010_A_swine_Wenzendorf_4584_2005_H1N1_1C.2.1
MK364871_A_swine_Kneheim_3066_2004_H1N1_1C.2.1
MK364962_A_swine_Wenzendorf_4353_2005_H1N1_1C.2.1
MK365303_A_swine_Bad_Wuennenberg_6005_2007_H1N1_1C.2.1
MK364879_A_swine_Laer_IDT3893_2005_H1N1_1C.2.1
MK365743_A_swine_Bartenshagen_9002_2009_H1N1_1C.2.1
MK365218_A_swine_Bad_Griesbach_IDT5604_2006_H1N1_1C.2.1
MK365233_A_swine_Rotthalmuenster_5616_2006_H1N1_1C.2.1
MK365241_A_swine_Feldbach_5625_2006_H1N1_1C.2.1
MK365319_A_swine_Schmallenberg_6049_2007_H1N1_1C.2.1

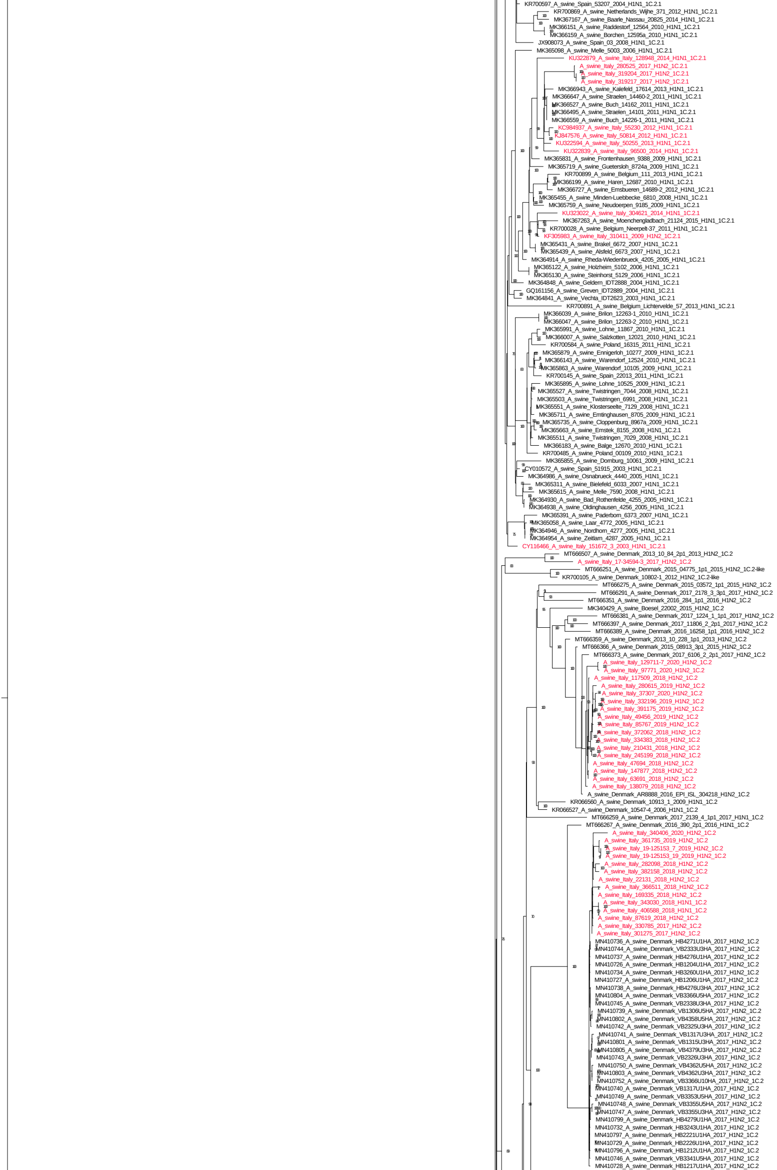
KR700597_A_swine_Spain_53207_2004_H1N1_1C.2.1
KR700869_A_swine_Netherlands_Wijhe_371_2012_H1N1_1C.2.1
MK367167_A_swine_Baarle_Nassau_20825_2014_H1N1_1C.2.1
MK366151_A_swine_Raddestorf_12564_2010_H1N1_1C.2.1
MK366159_A_swine_Borchen_12595a_2010_H1N1_1C.2.1
JX908073_A_swine_Spain_03_2008_H1N1_1C.2.1
MK365098_A_swine_Melle_5003_2006_H1N1_1C.2.1
KU322879_A_swine_Italy_128948_2014_H1N1_1C.2.1
A_swine_Italy_280525_2017_H1N2_1C.2.1
A_swine_Italy_319204_2017_H1N2_1C.2.1
A_swine_Italy_319217_2017_H1N2_1C.2.1
MK366943_A_swine_Kalefeld_17614_2013_H1N1_1C.2.1
MK366647_A_swine_Straelen_14460-2_2011_H1N1_1C.2.1
MK366527_A_swine_Buch_14162_2011_H1N1_1C.2.1
MK366495_A_swine_Straelen_14101_2011_H1N1_1C.2.1
MK366559_A_swine_Buch_14226-1_2011_H1N1_1C.2.1
KC984937_A_swine_Italy_55230_2012_H1N1_1C.2.1
KJ847576_A_swine_Italy_50814_2012_H1N1_1C.2.1
KU322594_A_swine_Italy_50255_2013_H1N1_1C.2.1
KU322839_A_swine_Italy_96500_2014_H1N1_1C.2.1
MK365831_A_swine_Frontenhausen_9388_2009_H1N1_1C.2.1
MK365719_A_swine_Guetersloh_8724a_2009_H1N1_1C.2.1
KR700899_A_swine_Belgium_111_2013_H1N1_1C.2.1
MK366199_A_swine_Haren_12687_2010_H1N1_1C.2.1
MK366727_A_swine_Emsbueren_14689-2_2012_H1N1_1C.2.1
MK365455_A_swine_Minden-Luebbecke_6810_2008_H1N1_1C.2.1
MK365759_A_swine_Neudoerpen_9185_2009_H1N1_1C.2.1
KU323022_A_swine_Italy_304621_2014_H1N1_1C.2.1
MK367263_A_swine_Moenchengladbach_21124_2015_H1N1_1C.2.1
KR700028_A_swine_Belgium_Neerpelt-37_2011_H1N1_1C.2.1
KF305983_A_swine_Italy_310411_2009_H1N2_1C.2.1
MK365431_A_swine_Brakel_6672_2007_H1N1_1C.2.1
MK365439_A_swine_Alsfeld_6673_2007_H1N1_1C.2.1
MK364914_A_swine_Rheda-Wiedenbrueck_4205_2005_H1N1_1C.2.1
MK365122_A_swine_Holzheim_5102_2006_H1N1_1C.2.1
MK365130_A_swine_Steinhorst_5129_2006_H1N1_1C.2.1
MK364848_A_swine_Geldern_IDT2888_2004_H1N1_1C.2.1
GQ161156_A_swine_Greven_IDT2889_2004_H1N1_1C.2.1
MK364841_A_swine_Vechta_IDT2623_2003_H1N1_1C.2.1
KR700891_A_swine_Belgium_Lichtervelde_57_2013_H1N1_1C.2.1
MK366039_A_swine_Brilon_12263-1_2010_H1N1_1C.2.1
MK366047_A_swine_Brilon_12263-2_2010_H1N1_1C.2.1
MK365991_A_swine_Lohne_11867_2010_H1N1_1C.2.1
MK366007_A_swine_Salzkotten_12021_2010_H1N1_1C.2.1
KR700584_A_swine_Poland_16315_2011_H1N1_1C.2.1
MK365879_A_swine_Ennigerloh_10277_2009_H1N1_1C.2.1
MK366143_A_swine_Warendorf_12524_2010_H1N1_1C.2.1
MK365863_A_swine_Warendorf_10105_2009_H1N1_1C.2.1
KR700145_A_swine_Spain_22013_2011_H1N1_1C.2.1
MK365895_A_swine_Lohne_10525_2009_H1N1_1C.2.1
MK365527_A_swine_Twistringen_7044_2008_H1N1_1C.2.1
MK365503_A_swine_Twistringen_6991_2008_H1N1_1C.2.1
MK365551_A_swine_Klosterseelte_7129_2008_H1N1_1C.2.1
MK365711_A_swine_Emtinghausen_8705_2009_H1N1_1C.2.1
MK365735_A_swine_Cloppenburg_8967a_2009_H1N1_1C.2.1
MK365663_A_swine_Emstek_8155_2008_H1N1_1C.2.1
MK365511_A_swine_Twistringen_7029_2008_H1N1_1C.2.1
MK366183_A_swine_Balge_12670_2010_H1N1_1C.2.1
KR700485_A_swine_Poland_00109_2010_H1N1_1C.2.1
MK365855_A_swine_Dornburg_10061_2009_H1N1_1C.2.1
CY010572_A_swine_Spain_51915_2003_H1N1_1C.2.1
MK364986_A_swine_Osnabrueck_4440_2005_H1N1_1C.2.1
MK365311_A_swine_Bielefeld_6033_2007_H1N1_1C.2.1
MK365615_A_swine_Melle_7590_2008_H1N1_1C.2.1
MK364930_A_swine_Bad_Rothenfelde_4255_2005_H1N1_1C.2.1
MK364938_A_swine_Oldinghausen_4256_2005_H1N1_1C.2.1
MK365391_A_swine_Paderborn_6373_2007_H1N1_1C.2.1
MK365058_A_swine_Laar_4772_2005_H1N1_1C.2.1
MK364946_A_swine_Nordhorn_4277_2005_H1N1_1C.2.1
MK364954_A_swine_Zeitlarn_4287_2005_H1N1_1C.2.1
CY116466_A_swine_Italy_151672_3_2003_H1N1_1C.2.1
MT666507_A_swine_Denmark_2013_10_84_2p1_2013_H1N2_1C.2
A_swine_Italy_17-34594-3_2017_H1N2_1C.2
MT666251_A_swine_Denmark_2015_04775_1p1_2015_H1N2_1C.2-like
KR700105_A_swine_Denmark_10802-1_2012_H1N2_1C.2-like
MT666275_A_swine_Denmark_2015_03572_1p1_2015_H1N2_1C.2
MT666291_A_swine_Denmark_2017_2178_3_3p1_2017_H1N2_1C.2
MT666351_A_swine_Denmark_2016_284_1p1_2016_H1N2_1C.2
MK340429_A_swine_Boesel_22002_2015_H1N2_1C.2
MT666381_A_swine_Denmark_2017_1224_1_1p1_2017_H1N2_1C.2
MT666397_A_swine_Denmark_2017_11806_2_2p1_2017_H1N2_1C.2
MT666389_A_swine_Denmark_2016_16258_1p1_2016_H1N2_1C.2
MT666359_A_swine_Denmark_2013_10_228_1p1_2013_H1N2_1C.2
MT666366_A_swine_Denmark_2015_08913_3p1_2015_H1N2_1C.2
MT666373_A_swine_Denmark_2017_6106_2_2p1_2017_H1N2_1C.2
A_swine_Italy_129711-7_2020_H1N2_1C.2
A_swine_Italy_97771_2020_H1N2_1C.2
A_swine_Italy_117509_2018_H1N2_1C.2
A_swine_Italy_280615_2019_H1N2_1C.2
A_swine_Italy_37307_2020_H1N2_1C.2
A_swine_Italy_332196_2019_H1N2_1C.2
A_swine_Italy_391175_2019_H1N2_1C.2
A_swine_Italy_49456_2019_H1N2_1C.2
A_swine_Italy_85767_2019_H1N2_1C.2
A_swine_Italy_372062_2018_H1N2_1C.2
A_swine_Italy_334383_2018_H1N2_1C.2
A_swine_Italy_210431_2018_H1N2_1C.2
A_swine_Italy_245199_2018_H1N2_1C.2
A_swine_Italy_47694_2018_H1N2_1C.2
A_swine_Italy_147877_2018_H1N2_1C.2
A_swine_Italy_63691_2018_H1N2_1C.2
A_swine_Italy_138079_2018_H1N2_1C.2
A_swine_Denmark_AR8888_2016_EPI_ISL_304218_H1N2_1C.2
KR066560_A_swine_Denmark_10913_1_2009_H1N1_1C.2
KR066527_A_swine_Denmark_10547-4_2006_H1N1_1C.2
MT666259_A_swine_Denmark_2017_2139_4_1p1_2017_H1N1_1C.2
MT666267_A_swine_Denmark_2016_390_2p1_2016_H1N1_1C.2
A_swine_Italy_340406_2020_H1N2_1C.2
A_swine_Italy_361735_2019_H1N2_1C.2
A_swine_Italy_19-125153_7_2019_H1N2_1C.2
A_swine_Italy_19-125153_19_2019_H1N2_1C.2
A_swine_Italy_282098_2018_H1N2_1C.2
A_swine_Italy_382158_2018_H1N2_1C.2
A_swine_Italy_22131_2018_H1N2_1C.2
A_swine_Italy_366511_2018_H1N2_1C.2
A_swine_Italy_169335_2018_H1N2_1C.2
A_swine_Italy_343030_2018_H1N1_1C.2
A_swine_Italy_406588_2018_H1N1_1C.2
A_swine_Italy_87619_2018_H1N2_1C.2
A_swine_Italy_330785_2017_H1N2_1C.2
A_swine_Italy_301275_2017_H1N2_1C.2
MN410736_A_swine_Denmark_HB4271U1HA_2017_H1N2_1C.2
MN410744_A_swine_Denmark_VB2333U3HA_2017_H1N2_1C.2
MN410737_A_swine_Denmark_HB4276U1HA_2017_H1N2_1C.2
MN410726_A_swine_Denmark_HB1204U1HA_2017_H1N2_1C.2
MN410734_A_swine_Denmark_HB3260U1HA_2017_H1N2_1C.2
MN410727_A_swine_Denmark_HB1206U1HA_2017_H1N2_1C.2
MN410738_A_swine_Denmark_HB4276U3HA_2017_H1N2_1C.2
MN410804_A_swine_Denmark_VB3366U5HA_2017_H1N2_1C.2
MN410745_A_swine_Denmark_VB2338U3HA_2017_H1N2_1C.2
MN410739_A_swine_Denmark_VB1306U5HA_2017_H1N2_1C.2
MN410802_A_swine_Denmark_VB4358U5HA_2017_H1N2_1C.2
MN410742_A_swine_Denmark_VB2325U3HA_2017_H1N2_1C.2
MN410741_A_swine_Denmark_VB1317U3HA_2017_H1N2_1C.2
MN410801_A_swine_Denmark_VB1315U3HA_2017_H1N2_1C.2
MN410805_A_swine_Denmark_VB4379U3HA_2017_H1N2_1C.2
MN410743_A_swine_Denmark_VB2326U3HA_2017_H1N2_1C.2
MN410750_A_swine_Denmark_VB4362U5HA_2017_H1N2_1C.2
MN410803_A_swine_Denmark_VB4362U3HA_2017_H1N2_1C.2
MN410752_A_swine_Denmark_VB3366U10HA_2017_H1N2_1C.2
MN410740_A_swine_Denmark_VB1317U5HA_2017_H1N2_1C.2
MN410749_A_swine_Denmark_VB3353U5HA_2017_H1N2_1C.2
MN410748_A_swine_Denmark_VB3355U5HA_2017_H1N2_1C.2
MN410747_A_swine_Denmark_VB3355U3HA_2017_H1N2_1C.2
MN410799_A_swine_Denmark_HB4279U1HA_2017_H1N2_1C.2
MN410732_A_swine_Denmark_HB3243U1HA_2017_H1N2_1C.2
MN410797_A_swine_Denmark_HB2221U1HA_2017_H1N2_1C.2
MN410729_A_swine_Denmark_HB2226U1HA_2017_H1N2_1C.2
MN410796_A_swine_Denmark_HB1212U1HA_2017_H1N2_1C.2
MN410746_A_swine_Denmark_VB3341U5HA_2017_H1N2_1C.2
MN410728_A_swine_Denmark_HB1217U1HA_2017_H1N2_1C.2

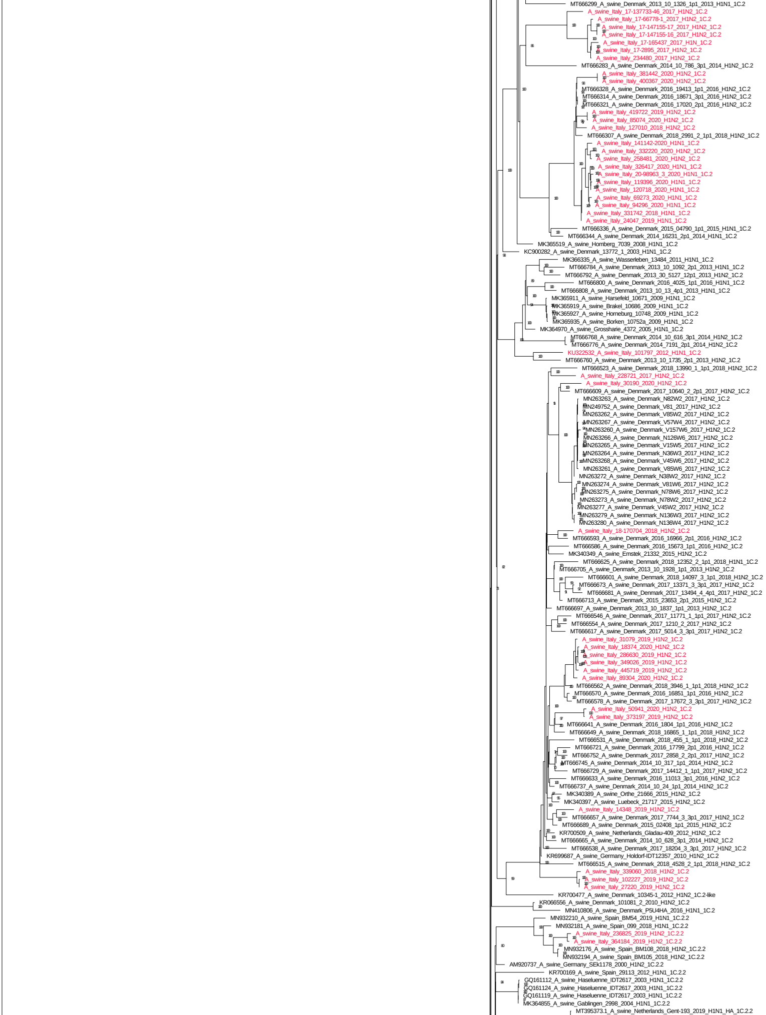
MT666299_A_swine_Denmark_2013_10_1326_1p1_2013_H1N1_1C.2
A_swine_Italy_17-137733-46_2017_H1N2_1C.2
A_swine_Italy_17-66778-1_2017_H1N2_1C.2
A_swine_Italy_17-147155-17_2017_H1N2_1C.2
A_swine_Italy_17-147155-16_2017_H1N2_1C.2
A_swine_Italy_17-165437_2017_H1N_1C.2
A_swine_Italy_17-2895_2017_H1N2_1C.2
A_swine_Italy_234480_2017_H1N2_1C.2
MT666283_A_swine_Denmark_2014_10_786_3p1_2014_H1N2_1C.2
A_swine_Italy_381442_2020_H1N2_1C.2
A_swine_Italy_400367_2020_H1N2_1C.2
MT666328_A_swine_Denmark_2016_19413_1p1_2016_H1N2_1C.2
MT666314_A_swine_Denmark_2016_18671_3p1_2016_H1N2_1C.2
MT666321_A_swine_Denmark_2016_17020_2p1_2016_H1N2_1C.2
A_swine_Italy_419722_2019_H1N2_1C.2
A_swine_Italy_85074_2020_H1N2_1C.2
A_swine_Italy_127010_2018_H1N2_1C.2
MT666307_A_swine_Denmark_2018_2991_2_1p1_2018_H1N2_1C.2
A_swine_Italy_141142-2020_H1N1_1C.2
A_swine_Italy_332220_2020_H1N2_1C.2
A_swine_Italy_258481_2020_H1N2_1C.2
A_swine_Italy_326417_2020_H1N1_1C.2
A_swine_Italy_20-98963_3_2020_H1N1_1C.2
A_swine_Italy_119396_2020_H1N1_1C.2
A_swine_Italy_120718_2020_H1N1_1C.2
A_swine_Italy_69273_2020_H1N1_1C.2
A_swine_Italy_94296_2020_H1N1_1C.2
A_swine_Italy_331742_2018_H1N1_1C.2
A_swine_Italy_24047_2019_H1N1_1C.2
MT666336_A_swine_Denmark_2015_04790_1p1_2015_H1N1_1C.2
MT666344_A_swine_Denmark_2014_16231_2p1_2014_H1N1_1C.2
MK365519_A_swine_Homberg_7039_2008_H1N1_1C.2
KC900282_A_swine_Denmark_13772_1_2003_H1N1_1C.2
MK366335_A_swine_Wasserleben_13484_2011_H1N1_1C.2
MT666784_A_swine_Denmark_2013_10_1092_2p1_2013_H1N1_1C.2
MT666792_A_swine_Denmark_2013_30_5127_12p1_2013_H1N2_1C.2
MT666800_A_swine_Denmark_2016_4025_1p1_2016_H1N1_1C.2
MT666808_A_swine_Denmark_2013_10_13_4p1_2013_H1N1_1C.2
MK365911_A_swine_Harsefeld_10671_2009_H1N1_1C.2
MK365919_A_swine_Brakel_10686_2009_H1N1_1C.2
MK365927_A_swine_Horneburg_10748_2009_H1N1_1C.2
MK365935_A_swine_Borken_10752a_2009_H1N1_1C.2
MK364970_A_swine_Grossharie_4372_2005_H1N1_1C.2
MT666768_A_swine_Denmark_2014_10_616_3p1_2014_H1N2_1C.2
MT666776_A_swine_Denmark_2014_7191_2p1_2014_H1N2_1C.2
KU322532_A_swine_Italy_101797_2012_H1N1_1C.2
MT666760_A_swine_Denmark_2013_10_1735_2p1_2013_H1N2_1C.2
MT666523_A_swine_Denmark_2018_13990_1_1p1_2018_H1N2_1C.2
A_swine_Italy_228721_2017_H1N2_1C.2
A_swine_Italy_30190_2020_H1N2_1C.2
MT666609_A_swine_Denmark_2017_10640_2_2p1_2017_H1N2_1C.2
MN263263_A_swine_Denmark_N82W2_2017_H1N2_1C.2
MN249752_A_swine_Denmark_V81_2017_H1N2_1C.2
MN263262_A_swine_Denmark_V85W2_2017_H1N2_1C.2
MN263267_A_swine_Denmark_V57W4_2017_H1N2_1C.2
MN263260_A_swine_Denmark_V157W6_2017_H1N2_1C.2
MN263266_A_swine_Denmark_N126W6_2017_H1N2_1C.2
MN263265_A_swine_Denmark_V15W5_2017_H1N2_1C.2
MN263264_A_swine_Denmark_N36W3_2017_H1N2_1C.2
MN263268_A_swine_Denmark_V45W6_2017_H1N2_1C.2
MN263261_A_swine_Denmark_V85W6_2017_H1N2_1C.2
MN263272_A_swine_Denmark_N38W2_2017_H1N2_1C.2
MN263274_A_swine_Denmark_V81W6_2017_H1N2_1C.2
MN263275_A_swine_Denmark_N78W6_2017_H1N2_1C.2
MN263273_A_swine_Denmark_N78W2_2017_H1N2_1C.2
MN263277_A_swine_Denmark_V45W2_2017_H1N2_1C.2
MN263279_A_swine_Denmark_N136W3_2017_H1N2_1C.2
MN263280_A_swine_Denmark_N136W4_2017_H1N2_1C.2
A_swine_Italy_18-170704_2018_H1N2_1C.2
MT666593_A_swine_Denmark_2016_16966_2p1_2016_H1N2_1C.2
MT666586_A_swine_Denmark_2016_15673_1p1_2016_H1N2_1C.2
MK340349_A_swine_Emstek_21332_2015_H1N2_1C.2
MT666625_A_swine_Denmark_2018_12352_2_1p1_2018_H1N1_1C.2
MT666705_A_swine_Denmark_2013_10_1928_1p1_2013_H1N2_1C.2
MT666601_A_swine_Denmark_2018_14097_3_1p1_2018_H1N2_1C.2
MT666673_A_swine_Denmark_2017_13371_3_3p1_2017_H1N2_1C.2
MT666681_A_swine_Denmark_2017_13494_4_4p1_2017_H1N2_1C.2
MT666713_A_swine_Denmark_2015_23653_2p1_2015_H1N2_1C.2
MT666697_A_swine_Denmark_2013_10_1837_1p1_2013_H1N2_1C.2
MT666546_A_swine_Denmark_2017_11771_1_1p1_2017_H1N2_1C.2
MT666554_A_swine_Denmark_2017_1210_2_2017_H1N2_1C.2
MT666617_A_swine_Denmark_2017_5014_3_3p1_2017_H1N2_1C.2
A_swine_Italy_31079_2019_H1N2_1C.2
A_swine_Italy_18374_2020_H1N2_1C.2
A_swine_Italy_286630_2019_H1N2_1C.2
A_swine_Italy_349026_2019_H1N2_1C.2
A_swine_Italy_445719_2019_H1N2_1C.2
A_swine_Italy_89304_2020_H1N2_1C.2
MT666562_A_swine_Denmark_2018_3946_1_1p1_2018_H1N2_1C.2
MT666570_A_swine_Denmark_2016_16851_1p1_2016_H1N2_1C.2
MT666578_A_swine_Denmark_2017_17672_3_3p1_2017_H1N2_1C.2
A_swine_Italy_50941_2020_H1N2_1C.2
A_swine_Italy_373197_2019_H1N2_1C.2
MT666641_A_swine_Denmark_2016_1804_1p1_2016_H1N2_1C.2
MT666649_A_swine_Denmark_2018_16865_1_1p1_2018_H1N2_1C.2
MT666531_A_swine_Denmark_2018_455_1_1p1_2018_H1N2_1C.2
MT666721_A_swine_Denmark_2016_17799_2p1_2016_H1N2_1C.2
MT666752_A_swine_Denmark_2017_2858_2_2p1_2017_H1N2_1C.2
MT666745_A_swine_Denmark_2014_10_317_1p1_2014_H1N2_1C.2
MT666729_A_swine_Denmark_2017_14412_1_1p1_2017_H1N2_1C.2
MT666633_A_swine_Denmark_2016_11013_3p1_2016_H1N2_1C.2
MT666737_A_swine_Denmark_2014_10_24_1p1_2014_H1N2_1C.2
MK340389_A_swine_Orthe_21666_2015_H1N2_1C.2
MK340397_A_swine_Luebeck_21717_2015_H1N2_1C.2
A_swine_Italy_14348_2019_H1N2_1C.2
MT666657_A_swine_Denmark_2017_7744_3_3p1_2017_H1N2_1C.2
MT666689_A_swine_Denmark_2015_02408_1p1_2015_H1N2_1C.2
KR700509_A_swine_Netherlands_Gladau-409_2012_H1N2_1C.2
MT666665_A_swine_Denmark_2014_10_628_3p1_2014_H1N2_1C.2
MT666538_A_swine_Denmark_2017_18204_3_3p1_2017_H1N2_1C.2
KR699687_A_swine_Germany_Holdorf-IDT12357_2010_H1N2_1C.2
MT666515_A_swine_Denmark_2018_4528_2_1p1_2018_H1N2_1C.2
A_swine_Italy_339060_2018_H1N2_1C.2
A_swine_Italy_102227_2019_H1N2_1C.2
A_swine_Italy_27220_2019_H1N2_1C.2
KR700477_A_swine_Denmark_10345-1_2012_H1N2_1C.2-like
KR066556_A_swine_Denmark_101081_2_2010_H1N2_1C.2
MN410806_A_swine_Denmark_P5U4HA_2016_H1N1_1C.2
MN932210_A_swine_Spain_BM54_2019_H1N1_1C.2.2
MN932181_A_swine_Spain_099_2018_H1N1_1C.2.2
A_swine_Italy_236825_2019_H1N2_1C.2.2
A_swine_Italy_364184_2019_H1N2_1C.2.2
MN932176_A_swine_Spain_BM108_2018_H1N2_1C.2.2
MN932194_A_swine_Spain_BM105_2018_H1N2_1C.2.2
AM920737_A_swine_Germany_SEk1178_2000_H1N2_1C.2.2
KR700169_A_swine_Spain_29113_2012_H1N1_1C.2.2
GQ161112_A_swine_Haseluenne_IDT2617_2003_H1N1_1C.2.2
GQ161124_A_swine_Haseluenne_IDT2617_2003_H1N1_1C.2.2
GQ161119_A_swine_Haseluenne_IDT2617_2003_H1N1_1C.2.2
MK364855_A_swine_Gablingen_2998_2004_H1N1_1C.2.2
MT395373.1_A_swine_Netherlands_Gent-193_2019_H1N1_HA_1C.2.2

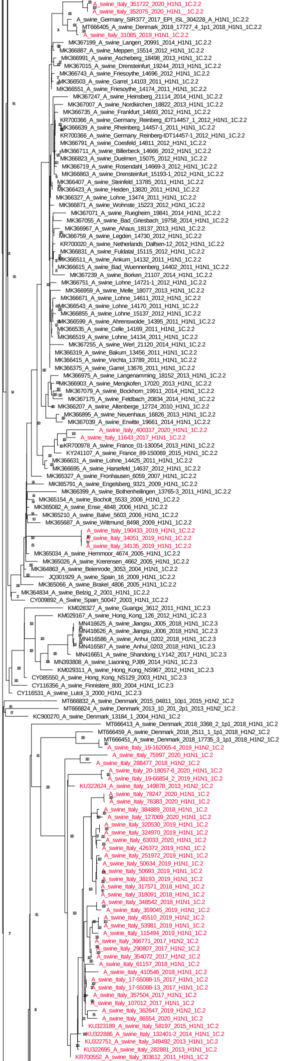
A_swine_Italy_351722_2020_H1N1_1C.2.2
A_swine_Italy_352075_2020_H1N1_1C.2.2
A_swine_Germany_SIR377_2017_EPI_ISL_304228_A_H1N1_1C.2.2
MT666405_A_swine_Denmark_2018_17727_4_1p1_2018_H1N1_1C.2.2
A_swine_Italy_31085_2019_H1N1_1C.2.2
MK367199_A_swine_Langen_20991_2014_H1N1_1C.2.2
MK366887_A_swine_Meppen_15514_2012_H1N1_1C.2.2
MK366991_A_swine_Ascheberg_18498_2013_H1N1_1C.2.2
MK367015_A_swine_Drensteinfurt_19244_2013_H1N1_1C.2.2
MK366743_A_swine_Friesoythe_14696_2012_H1N1_1C.2.2
MK366503_A_swine_Garrel_14103_2011_H1N1_1C.2.2
MK366551_A_swine_Friesoythe_14174_2011_H1N1_1C.2.2
MK367247_A_swine_Heinsberg_21114_2014_H1N1_1C.2.2
MK367007_A_swine_Nordkirchen_18822_2013_H1N1_1C.2.2
MK366735_A_swine_Frankfurt_14693_2012_H1N1_1C.2.2
KR700366_A_swine_Germany_Reinberg_IDT14457_1_2012_H1N1_1C.2.2
MK366639_A_swine_Rheinberg_14457-1_2011_H1N1_1C.2.2
KR700366_A_swine_Germany_Reinberg-IDT14457-1_2012_H1N1_1C.2.2
MK366791_A_swine_Coesfeld_14811_2012_H1N1_1C.2.2
MK366711_A_swine_Billerbeck_14666_2012_H1N1_1C.2.2
MK366823_A_swine_Duelmen_15075_2012_H1N1_1C.2.2
MK366719_A_swine_Rosendahl_14669-3_2012_H1N1_1C.2.2
MK366863_A_swine_Drensteinfurt_15193-1_2012_H1N1_1C.2.2
MK366407_A_swine_Steinfeld_13785_2011_H1N1_1C.2.2
MK366423_A_swine_Heiden_13820_2011_H1N1_1C.2.2
MK366327_A_swine_Lohne_13474_2011_H1N1_1C.2.2
MK366871_A_swine_Wohnste_15223_2012_H1N1_1C.2.2
MK367071_A_swine_Ruegheim_19841_2014_H1N1_1C.2.2
MK367055_A_swine_Bad_Griesbach_19758_2014_H1N1_1C.2.2
MK366967_A_swine_Ahaus_18137_2013_H1N1_1C.2.2
MK366759_A_swine_Legden_14730_2012_H1N1_1C.2.2
KR700020_A_swine_Netherlands_Dalfsen-12_2012_H1N1_1C.2.2
MK366831_A_swine_Fuldatal_15115_2012_H1N1_1C.2.2
MK366511_A_swine_Ankum_14132_2011_H1N1_1C.2.2
MK366615_A_swine_Bad_Wuennenberg_14402_2011_H1N1_1C.2.2
MK367239_A_swine_Borken_21107_2014_H1N1_1C.2.2
MK366751_A_swine_Lohne_14721-1_2012_H1N1_1C.2.2
MK366959_A_swine_Melle_18077_2013_H1N1_1C.2.2
MK366671_A_swine_Lohne_14611_2012_H1N1_1C.2.2
MK366543_A_swine_Lohne_14170_2011_H1N1_1C.2.2
MK366855_A_swine_Lohne_15137_2012_H1N1_1C.2.2
MK366599_A_swine_Ahrenswolde_14395_2011_H1N1_1C.2.2
MK366535_A_swine_Celle_14169_2011_H1N1_1C.2.2
MK366519_A_swine_Lohne_14134_2011_H1N1_1C.2.2
MK367255_A_swine_Werl_21120_2014_H1N1_1C.2.2
MK366319_A_swine_Bakum_13456_2011_H1N1_1C.2.2
MK366415_A_swine_Vechta_13789_2011_H1N1_1C.2.2
MK366375_A_swine_Garrel_13676_2011_H1N1_1C.2.2
MK366975_A_swine_Langenamming_18152_2013_H1N1_1C.2.2
MK366903_A_swine_Mengkofen_17020_2013_H1N1_1C.2.2
MK367079_A_swine_Bockhorn_19911_2014_H1N1_1C.2.2
MK367175_A_swine_Feldbach_20834_2014_H1N1_1C.2.2
MK366207_A_swine_Altenberge_12724_2010_H1N1_1C.2.2
MK366895_A_swine_Neuenhaus_16826_2013_H1N1_1C.2.2
MK367039_A_swine_Erwitte_19661_2014_H1N1_1C.2.2
A_swine_Italy_400317_2020_H1N1_1C.2.2
A_swine_Italy_11643_2017_H1N1_1C.2.2
KR700978_A_swine_France_01-130054_2013_H1N1_1C.2.2
KY241107_A_swine_France_89-150069_2015_H1N1_1C.2.2
MK366631_A_swine_Lohne_14425_2011_H1N1_1C.2.2
MK366695_A_swine_Harsefeld_14637_2012_H1N1_1C.2.2
MK365327_A_swine_Fronhausen_6059_2007_H1N1_1C.2.2
MK365791_A_swine_Engelsberg_9321_2009_H1N1_1C.2.2
MK366399_A_swine_Bothenheilingen_13765-3_2011_H1N1_1C.2.2
MK365154_A_swine_Bocholt_5533_2006_H1N1_1C.2.2
MK365082_A_swine_Ense_4848_2006_H1N1_1C.2.2
MK365210_A_swine_Balve_5603_2006_H1N1_1C.2.2
MK365687_A_swine_Wittmund_8498_2009_H1N1_1C.2.2
A_swine_Italy_190433_2019_H1N1_1C.2.2
A_swine_Italy_34051_2019_H1N1_1C.2.2
A_swine_Italy_34135_2019_H1N1_1C.2.2
MK365034_A_swine_Hemmoor_4674_2005_H1N1_1C.2.2
MK365026_A_swine_Krerensen_4662_2005_H1N1_1C.2.2
MK364863_A_swine_Beienrode_3053_2004_H1N1_1C.2.2
JQ301929_A_swine_Spain_16_2009_H1N1_1C.2.2
MK365066_A_swine_Brakel_4806_2005_H1N1_1C.2.2
MK364834_A_swine_Belzig_2_2001_H1N1_1C.2.2
CY009892_A_Swine_Spain_50047_2003_H1N1_1C.2.2
KM028327_A_swine_Guangxi_3612_2011_H1N1_1C.2.3
KM029167_A_swine_Hong_Kong_126_2012_H1N1_1C.2.3
MN416625_A_swine_Jiangsu_J005_2018_H1N1_1C.2.3
MN416626_A_swine_Jiangsu_J006_2018_H1N1_1C.2.3
MN416586_A_swine_Anhui_0202_2018_H1N1_1C.2.3
MN416587_A_swine_Anhui_0203_2018_H1N1_1C.2.3
MN416651_A_swine_Shandong_LY142_2017_H1N1_1C.2.3
MN393808_A_swine_Liaoning_PJ89_2014_H1N1_1C.2.3
KM029311_A_swine_Hong_Kong_NS967_2012_H1N1_1C.2.3
CY085550_A_swine_Hong_Kong_NS129_2003_H1N1_1C.2.3
CY116356_A_swine_Finnistere_800_2004_H1N1_1C.2.3
CY116531_A_swine_Lutol_3_2000_H1N1_1C.2.3
MT666832_A_swine_Denmark_2015_04811_10p1_2015_H1N2_1C.2
MT666824_A_swine_Denmark_2013_10_201_2p1_2013_H1N2_1C.2
KC900270_A_swine_Denmark_13184_1_2004_H1N1_1C.2
MT666413_A_swine_Denmark_2018_3368_2_1p1_2018_H1N1_1C.2
MT666459_A_swine_Denmark_2018_2511_1_1p1_2018_H1N2_1C.2
MT666451_A_swine_Denmark_2018_17735_3_1p1_2018_H1N2_1C.2
A_swine_Italy_19-162065-4_2019_H1N2_1C.2
A_swine_Italy_75997_2020_H1N1_1C.2
A_swine_Italy_288477_2018_H1N2_1C.2
A_swine_Italy_20-18057-6_2020_H1N1_1C.2
A_swine_Italy_19-66854_2_2019_H1N1_1C.2
KU322624_A_swine_Italy_149878_2013_H1N2_1C.2
A_swine_Italy_78247_2020_H1N1_1C.2
A_swine_Italy_78383_2020_H1N1_1C.2
A_swine_Italy_384889_2018_H1N1_1C.2
A_swine_Italy_127069_2020_H1N1_1C.2
A_swine_Italy_320530_2019_H1N1_1C.2
A_swine_Italy_324970_2019_H1N1_1C.2
A_swine_Italy_63033_2020_H1N1_1C.2
A_swine_Italy_426372_2019_H1N1_1C.2
A_swine_Italy_251972_2019_H1N1_1C.2
A_swine_Italy_50634_2019_H1N1_1C.2
A_swine_Italy_50693_2019_H1N1_1C.2
A_swine_Italy_38193_2019_H1N1_1C.2
A_swine_Italy_317571_2018_H1N1_1C.2
A_swine_Italy_318091_2018_H1N1_1C.2
A_swine_Italy_348542_2018_H1N1_1C.2
A_swine_Italy_359045_2019_H1N1_1C.2
A_swine_Italy_45510_2019_H1N2_1C.2
A_swine_Italy_53981_2019_H1N1_1C.2
A_swine_Italy_115494_2019_H1N1_1C.2
A_swine_Italy_366771_2017_H1N2_1C.2
A_swine_Italy_290807_2017_H1N2_1C.2
A_swine_Italy_354072_2017_H1N2_1C.2
A_swine_Italy_61157_2018_H1N1_1C.2
A_swine_Italy_410546_2018_H1N1_1C.2
A_swine_Italy_17-55088-15_2017_H1N1_1C.2
A_swine_Italy_17-55088-13_2017_H1N1_1C.2
A_swine_Italy_357504_2017_H1N1_1C.2
A_swine_Italy_107012_2017_H1N1_1C.2
A_swine_Italy_362647_2019_H1N2_1C.2
A_swine_Italy_86554_2020_H1N1_1C.2
KU323189_A_swine_Italy_58197_2015_H1N1_1C.2
KU322886_A_swine_Italy_132401-2_2014_H1N1_1C.2
KU322751_A_swine_Italy_349492_2013_H1N1_1C.2
KU322695_A_swine_Italy_282881_2013_H1N1_1C.2
KR700552_A_swine_Italy_303612_2011_H1N1_1C.2

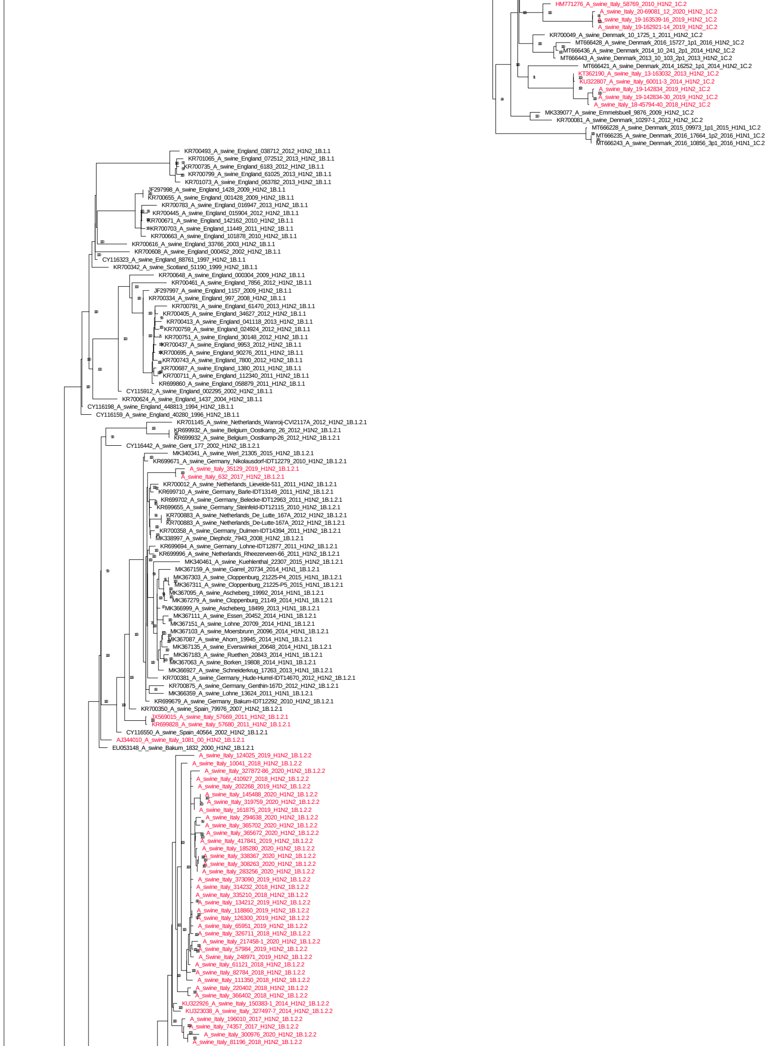

HM771276_A_swine_Italy_58769_2010_H1N2_1C.2
A_swine_Italy_20-69081_12_2020_H1N2_1C.2
A_swine_Italy_19-163539-16_2019_H1N2_1C.2
A_swine_Italy_19-162921-14_2019_H1N2_1C.2
KR700049_A_swine_Denmark_10_1725_1_2011_H1N2_1C.2
MT666428_A_swine_Denmark_2016_15727_1p1_2016_H1N2_1C.2
MT666436_A_swine_Denmark_2014_10_241_2p1_2014_H1N2_1C.2
MT666443_A_swine_Denmark_2013_10_103_2p1_2013_H1N2_1C.2
MT666421_A_swine_Denmark_2014_16252_1p1_2014_H1N2_1C.2
KT362190_A_swine_Italy_13-163032_2013_H1N2_1C.2
KU322807_A_swine_Italy_60011-3_2014_H1N2_1C.2
A_swine_Italy_19-142834_2019_H1N2_1C.2
A_swine_Italy_19-142834-30_2019_H1N2_1C.2
A_swine_Italy_18-45794-40_2018_H1N2_1C.2
MK339077_A_swine_Emmelsbuell_9876_2009_H1N2_1C.2
KR700081_A_swine_Denmark_10297-1_2012_H1N2_1C.2
MT666228_A_swine_Denmark_2015_09973_1p1_2015_H1N1_1C.2
MT666235_A_swine_Denmark_2016_17664_1p2_2016_H1N1_1C.2
MT666243_A_swine_Denmark_2016_10856_3p1_2016_H1N1_1C.2
KR700493_A_swine_England_038712_2012_H1N2_1B.1.1
KR701065_A_swine_England_072512_2013_H1N2_1B.1.1
KR700735_A_swine_England_6183_2012_H1N2_1B.1.1
KR700799_A_swine_England_61025_2013_H1N2_1B.1.1
KR701073_A_swine_England_063782_2013_H1N2_1B.1.1
JF297998_A_swine_England_1428_2009_H1N2_1B.1.1
KR700655_A_swine_England_001428_2009_H1N2_1B.1.1
KR700783_A_swine_England_016947_2013_H1N2_1B.1.1
KR700445_A_swine_England_015904_2012_H1N2_1B.1.1
KR700671_A_swine_England_142162_2010_H1N2_1B.1.1
KR700703_A_swine_England_11449_2011_H1N2_1B.1.1
KR700663_A_swine_England_101878_2010_H1N2_1B.1.1
KR700616_A_swine_England_33766_2003_H1N2_1B.1.1
KR700608_A_swine_England_000452_2002_H1N2_1B.1.1
CY116323_A_swine_England_88761_1997_H1N2_1B.1.1
KR700342_A_swine_Scotland_51190_1999_H1N2_1B.1.1
KR700648_A_swine_England_000304_2009_H1N2_1B.1.1
KR700461_A_swine_England_7856_2012_H1N2_1B.1.1
JF297997_A_swine_England_1157_2009_H1N2_1B.1.1
KR700334_A_swine_England_997_2008_H1N2_1B.1.1
KR700791_A_swine_England_61470_2013_H1N2_1B.1.1
KR700405_A_swine_England_34627_2012_H1N2_1B.1.1
KR700413_A_swine_England_041118_2013_H1N2_1B.1.1
KR700759_A_swine_England_024924_2012_H1N2_1B.1.1
KR700751_A_swine_England_30148_2012_H1N2_1B.1.1
KR700437_A_swine_England_9953_2012_H1N2_1B.1.1
KR700695_A_swine_England_90276_2011_H1N2_1B.1.1
KR700743_A_swine_England_7800_2012_H1N2_1B.1.1
KR700687_A_swine_England_1380_2011_H1N2_1B.1.1
KR700711_A_swine_England_112340_2011_H1N2_1B.1.1
KR699860_A_swine_England_058879_2011_H1N2_1B.1.1
CY115912_A_swine_England_002295_2002_H1N2_1B.1.1
KR700624_A_swine_England_1437_2004_H1N2_1B.1.1
CY116198_A_swine_England_448813_1994_H1N2_1B.1.1
CY116159_A_swine_England_40280_1996_H1N2_1B.1.1
KR701145_A_swine_Netherlands_Wanroij-CVI2117A_2012_H1N2_1B.1.2.1
KR699932_A_swine_Belgium_Oostkamp_26_2012_H1N2_1B.1.2.1
KR699932_A_swine_Belgium_Oostkamp-26_2012_H1N2_1B.1.2.1
CY116442_A_swine_Gent_177_2002_H1N2_1B.1.2.1
MK340341_A_swine_Werl_21305_2015_H1N2_1B.1.2.1
KR699671_A_swine_Germany_Nikolausdorf-IDT12279_2010_H1N2_1B.1.2.1
A_swine_Italy_35129_2019_H1N2_1B.1.2.1
A_swine_Italy_632_2017_H1N2_1B.1.2.1
KR700012_A_swine_Netherlands_Lievelde-511_2011_H1N2_1B.1.2.1
KR699710_A_swine_Germany_Barle-IDT13149_2011_H1N2_1B.1.2.1
KR699702_A_swine_Germany_Belecke-IDT12963_2011_H1N2_1B.1.2.1
KR699655_A_swine_Germany_Steinfeld-IDT12115_2010_H1N2_1B.1.2.1
KR700883_A_swine_Netherlands_De_Lutte_167A_2012_H1N2_1B.1.2.1
KR700883_A_swine_Netherlands_De-Lutte-167A_2012_H1N2_1B.1.2.1
KR700358_A_swine_Germany_Dulmen-IDT14394_2011_H1N2_1B.1.2.1
MK338997_A_swine_Diepholz_7943_2008_H1N2_1B.1.2.1
KR699694_A_swine_Germany_Lohne-IDT12877_2011_H1N2_1B.1.2.1
KR699996_A_swine_Netherlands_Rheezerveen-66_2011_H1N2_1B.1.2.1
MK340461_A_swine_Kuehlenthal_22307_2015_H1N2_1B.1.2.1
MK367159_A_swine_Garrel_20734_2014_H1N1_1B.1.2.1
MK367303_A_swine_Cloppenburg_21225-P4_2015_H1N1_1B.1.2.1
MK367311_A_swine_Cloppenburg_21225-P5_2015_H1N1_1B.1.2.1
MK367095_A_swine_Ascheberg_19992_2014_H1N1_1B.1.2.1
MK367279_A_swine_Cloppenburg_21149_2014_H1N1_1B.1.2.1
MK366999_A_swine_Ascheberg_18499_2013_H1N1_1B.1.2.1
MK367111_A_swine_Essen_20452_2014_H1N1_1B.1.2.1
MK367151_A_swine_Lohne_20709_2014_H1N1_1B.1.2.1
MK367103_A_swine_Moersbrunn_20096_2014_H1N1_1B.1.2.1
MK367087_A_swine_Ahorn_19945_2014_H1N1_1B.1.2.1
MK367135_A_swine_Everswinkel_20648_2014_H1N1_1B.1.2.1
MK367183_A_swine_Ruethen_20843_2014_H1N1_1B.1.2.1
MK367063_A_swine_Borken_19808_2014_H1N1_1B.1.2.1
MK366927_A_swine_Schneiderkrug_17263_2013_H1N1_1B.1.2.1
KR700381_A_swine_Germany_Hude-Hurrel-IDT14670_2012_H1N2_1B.1.2.1
KR700875_A_swine_Germany_Genthin-167D_2012_H1N2_1B.1.2.1
MK366359_A_swine_Lohne_13624_2011_H1N1_1B.1.2.1
KR699679_A_swine_Germany_Bakum-IDT12292_2010_H1N2_1B.1.2.1
KR700350_A_swine_Spain_79976_2007_H1N2_1B.1.2.1
JX569015_A_swine_Italy_57669_2011_H1N2_1B.1.2.1
KR699828_A_swine_Italy_57680_2011_H1N2_1B.1.2.1
CY116550_A_swine_Spain_40564_2002_H1N2_1B.1.2.1
AJ344010_A_swine_Italy_1081_00_H1N2_1B.1.2.1
EU053148_A_swine_Bakum_1832_2000_H1N2_1B.1.2.1
A_swine_Italy_124025_2019_H1N2_1B.1.2.2
A_swine_Italy_10041_2018_H1N2_1B.1.2.2
A_swine_Italy_327872-86_2020_H1N2_1B.1.2.2
A_swine_Italy_410927_2018_H1N2_1B.1.2.2
A_swine_Italy_202268_2019_H1N2_1B.1.2.2
A_swine_Italy_145488_2020_H1N2_1B.1.2.2
A_swine_Italy_319759_2020_H1N2_1B.1.2.2
A_swine_Italy_161875_2019_H1N2_1B.1.2.2
A_swine_Italy_294638_2020_H1N2_1B.1.2.2
A_swine_Italy_365702_2020_H1N2_1B.1.2.2
A_swine_Italy_365672_2020_H1N2_1B.1.2.2
A_swine_Italy_417841_2019_H1N2_1B.1.2.2
A_swine_Italy_185280_2020_H1N2_1B.1.2.2
A_swine_Italy_338367_2020_H1N2_1B.1.2.2
A_swine_Italy_308263_2020_H1N2_1B.1.2.2
A_swine_Italy_283256_2020_H1N2_1B.1.2.2
A_swine_Italy_373090_2019_H1N2_1B.1.2.2
A_swine_Italy_314232_2018_H1N2_1B.1.2.2
A_swine_Italy_335210_2018_H1N2_1B.1.2.2
A_swine_Italy_134212_2019_H1N2_1B.1.2.2
A_swine_Italy_118860_2019_H1N2_1B.1.2.2
A_swine_Italy_126300_2019_H1N2_1B.1.2.2
A_swine_Italy_65951_2019_H1N2_1B.1.2.2
A_swine_Italy_326711_2018_H1N2_1B.1.2.2
A_swine_Italy_217458-1_2020_H1N2_1B.1.2.2
A_swine_Italy_57984_2019_H1N2_1B.1.2.2
A_Swine_Italy_248971_2019_H1N2_1B.1.2.2
A_swine_Italy_61121_2018_H1N2_1B.1.2.2
A_swine_Italy_82784_2018_H1N2_1B.1.2.2
A_swine_Italy_111350_2018_H1N2_1B.1.2.2
A_swine_Italy_220402_2018_H1N2_1B.1.2.2
A_swine_Italy_366402_2018_H1N2_1B.1.2.2
KU322926_A_swine_Italy_150383-1_2014_H1N2_1B.1.2.2
KU323038_A_swine_Italy_327497-7_2014_H1N2_1B.1.2.2
A_swine_Italy_196010_2017_H1N2_1B.1.2.2
A_swine_Italy_74357_2017_H1N2_1B.1.2.2
A_swine_Italy_300976_2020_H1N2_1B.1.2.2
A_swine_Italy_81196_2018_H1N2_1B.1.2.2

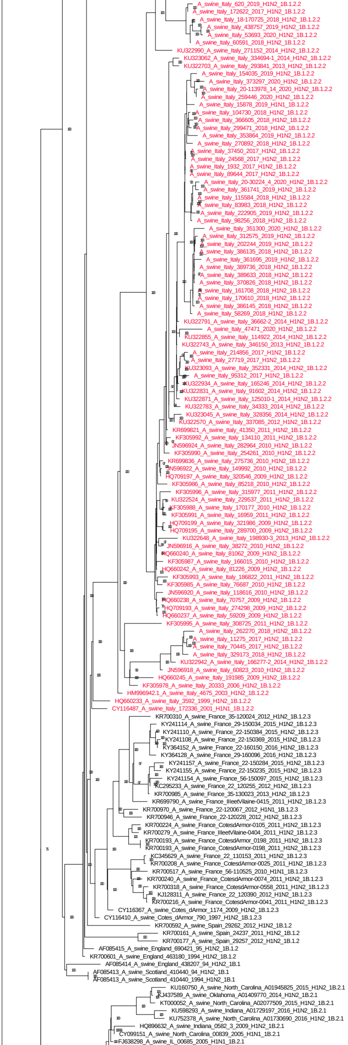

A_swine_Italy_620_2019_H1N2_1B.1.2.2
A_swine_Italy_172622_2017_H1N2_1B.1.2.2
A_swine_Italy_18-170725_2018_H1N2_1B.1.2.2
A_swine_Italy_438757_2019_H1N2_1B.1.2.2
A_swine_Italy_53693_2020_H1N2_1B.1.2.2
A_swine_Italy_60591_2018_H1N2_1B.1.2.2
KU322990_A_swine_Italy_271152_2014_H1N2_1B.1.2.2
KU323062_A_swine_Italy_334694-1_2014_H1N2_1B.1.2.2
KU322703_A_swine_Italy_293841_2013_H1N2_1B.1.2.2
A_swine_Italy_154035_2019_H1N2_1B.1.2.2
A_swine_Italy_373297_2020_H1N2_1B.1.2.2
A_swine_Italy_20-113978_14_2020_H1N2_1B.1.2.2
A_swine_Italy_259446_2020_H1N2_1B.1.2.2
A_swine_Italy_15878_2019_H1N1_1B.1.2.2
A_swine_Italy_104730_2018_H1N2_1B.1.2.2
A_swine_Italy_366605_2018_H1N2_1B.1.2.2
A_swine_Italy_299471_2018_H1N2_1B.1.2.2
A_swine_Italy_353864_2019_H1N2_1B.1.2.2
A_swine_Italy_270892_2018_H1N2_1B.1.2.2
A_swine_Italy_37450_2017_H1N2_1B.1.2.2
A_swine_Italy_24568_2017_H1N2_1B.1.2.2
A_swine_Italy_1932_2017_H1N2_1B.1.2.2
A_swine_Italy_89644_2017_H1N2_1B.1.2.2
A_swine_Italy_20-30224_4_2020_H1N2_1B.1.2.2
A_swine_Italy_361741_2019_H1N2_1B.1.2.2
A_swine_Italy_115584_2018_H1N2_1B.1.2.2
A_swine_Italy_83983_2018_H1N2_1B.1.2.2
A_swine_Italy_222905_2019_H1N2_1B.1.2.2
A_swine_Italy_98256_2018_H1N2_1B.1.2.2
A_swine_Italy_351300_2020_H1N2_1B.1.2.2
A_swine_Italy_312575_2019_H1N2_1B.1.2.2
A_swine_Italy_202244_2019_H1N2_1B.1.2.2
A_swine_Italy_386135_2018_H1N2_1B.1.2.2
A_swine_Italy_361695_2019_H1N2_1B.1.2.2
A_swine_Italy_389736_2018_H1N2_1B.1.2.2
A_swine_Italy_389633_2018_H1N2_1B.1.2.2
A_swine_Italy_370826_2018_H1N2_1B.1.2.2
A_swine_Italy_161708_2018_H1N2_1B.1.2.2
A_swine_Italy_170610_2018_H1N2_1B.1.2.2
A_swine_Italy_386145_2018_H1N2_1B.1.2.2
A_swine_Italy_58269_2018_H1N2_1B.1.2.2
KU322791_A_swine_Italy_36662-2_2014_H1N2_1B.1.2.2
A_swine_Italy_47471_2020_H1N2_1B.1.2.2
KU322855_A_swine_Italy_114922_2014_H1N2_1B.1.2.2
KU322743_A_swine_Italy_346150_2013_H1N2_1B.1.2.2
A_swine_Italy_214856_2017_H1N2_1B.1.2.2
A_swine_Italy_27719_2017_H1N2_1B.1.2.2
KU323093_A_swine_Italy_352331_2014_H1N2_1B.1.2.2
A_swine_Italy_95312_2017_H1N2_1B.1.2.2
KU322934_A_swine_Italy_165246_2014_H1N2_1B.1.2.2
KU322831_A_swine_Italy_91602_2014_H1N2_1B.1.2.2
KU322871_A_swine_Italy_125010-1_2014_H1N2_1B.1.2.2
KU322783_A_swine_Italy_34333_2014_H1N2_1B.1.2.2
KU323045_A_swine_Italy_328356_2014_H1N2_1B.1.2.2
KU322570_A_swine_Italy_337085_2012_H1N2_1B.1.2.2
KR699821_A_swine_Italy_41350_2011_H1N2_1B.1.2.2
KF305992_A_swine_Italy_134110_2011_H1N2_1B.1.2.2
JN596924_A_swine_Italy_282964_2010_H1N2_1B.1.2.2
KF305990_A_swine_Italy_254261_2010_H1N2_1B.1.2.2
KR699836_A_swine_Italy_275736_2010_H1N2_1B.1.2.2
JN596922_A_swine_Italy_149992_2010_H1N2_1B.1.2.2
HQ709197_A_swine_Italy_320546_2009_H1N2_1B.1.2.2
KF305986_A_swine_Italy_85218_2010_H1N2_1B.1.2.2
KF305996_A_swine_Italy_315977_2011_H1N2_1B.1.2.2
KU322524_A_swine_Italy_229537_2011_H1N2_1B.1.2.2
KF305988_A_swine_Italy_170177_2010_H1N2_1B.1.2.2
KF305991_A_swine_Italy_16959_2011_H1N2_1B.1.2.2
HQ709199_A_swine_Italy_321986_2009_H1N2_1B.1.2.2
HQ709195_A_swine_Italy_289700_2009_H1N2_1B.1.2.2
KU322648_A_swine_Italy_198930-3_2013_H1N2_1B.1.2.2
JN596916_A_swine_Italy_38272_2010_H1N2_1B.1.2.2
HQ660240_A_swine_Italy_81062_2009_H1N2_1B.1.2.2
KF305987_A_swine_Italy_166015_2010_H1N2_1B.1.2.2
HQ660242_A_swine_Italy_81226_2009_H1N2_1B.1.2.2
KF305993_A_swine_Italy_186822_2011_H1N2_1B.1.2.2
KF305985_A_swine_Italy_76687_2010_H1N2_1B.1.2.2
JN596920_A_swine_Italy_118616_2010_H1N2_1B.1.2.2
HQ660238_A_swine_Italy_70757_2009_H1N2_1B.1.2.2
HQ709193_A_swine_Italy_274298_2009_H1N2_1B.1.2.2
HQ660237_A_swine_Italy_59209_2009_H1N2_1B.1.2.2
KF305995_A_swine_Italy_308725_2011_H1N2_1B.1.2.2
A_swine_Italy_262270_2018_H1N2_1B.1.2.2
A_swine_Italy_11275_2017_H1N2_1B.1.2.2
A_swine_Italy_70445_2017_H1N2_1B.1.2.2
A_swine_Italy_329173_2018_H1N2_1B.1.2.2
KU322942_A_swine_Italy_166277-2_2014_H1N2_1B.1.2.2
JN596918_A_swine_Italy_60823_2010_H1N2_1B.1.2.2
HQ660245_A_swine_Italy_191985_2009_H1N2_1B.1.2.2
KF305978_A_swine_Italy_20333_2006_H1N2_1B.1.2.2
HM996942.1_A_swine_Italy_4675_2003_H1N2_1B.1.2.2
HQ660233_A_swine_Italy_3592_1999_H1N2_1B.1.2.2
CY116487_A_swine_Italy_172336_2001_H1N1_1B.1.2.2
KR700310_A_swine_France_35-120024_2012_H1N2_1B.1.2.3
KY241114_A_swine_France_29-150034_2015_H1N2_1B.1.2.3
KY241110_A_swine_France_22-150384_2015_H1N2_1B.1.2.3
KY241108_A_swine_France_22-150369_2015_H1N2_1B.1.2.3
KY364152_A_swine_France_22-160150_2016_H1N2_1B.1.2.3
KY364128_A_swine_France_29-160096_2016_H1N2_1B.1.2.3
KY241157_A_swine_France_22-150284_2015_H1N2_1B.1.2.3
KY241155_A_swine_France_22-150235_2015_H1N2_1B.1.2.3
KY241154_A_swine_France_56-150097_2015_H1N2_1B.1.2.3
KC295233_A_swine_France_22_120255_2012_H1N2_1B.1.2.3
KR700985_A_swine_France_35-130023_2013_H1N2_1B.1.2.3
KR699790_A_swine_France_IlleetVilaine-0415_2011_H1N2_1B.1.2.3
KR700970_A_swine_France_22-120067_2012_H1N1_1B.1.2.3
KR700946_A_swine_France_22-120228_2012_H1N2_1B.1.2.3
KR700224_A_swine_France_CotesdArmor-0105_2011_H1N2_1B.1.2.3
KR700279_A_swine_France_IlleetVilaine-0404_2011_H1N2_1B.1.2.3
KR700193_A_swine_France_CotesdArmor_0198_2011_H1N2_1B.1.2.3
KR700193_A_swine_France_CotesdArmor-0198_2011_H1N2_1B.1.2.3
KC345629_A_swine_France_22_110153_2011_H1N2_1B.1.2.3
KR700208_A_swine_France_CotesdArmor-0025_2011_H1N2_1B.1.2.3
KR700517_A_swine_France_56-110525_2010_H1N1_1B.1.2.3
KR700240_A_swine_France_CotesdArmor-0074_2011_H1N2_1B.1.2.3
KR700318_A_swine_France_CotesdArmor-0558_2011_H1N2_1B.1.2.3
KJ128311_A_swine_France_22_120390_2012_H1N2_1B.1.2.3
KR700216_A_swine_France_CotesdArmor-0041_2011_H1N2_1B.1.2.3
CY116367_A_swine_Cotes_dArmor_1174_2009_H1N2_1B.1.2.3
CY116410_A_swine_Cotes_dArmor_790_1997_H1N2_1B.1.2.3
KR700592_A_swine_Spain_29262_2012_H1N2_1B.1.2
KR700161_A_swine_Spain_24237_2011_H1N2_1B.1.2
KR700177_A_swine_Spain_29257_2012_H1N2_1B.1.2
AF085415_A_swine_England_690421_95_H1N2_1B.1.2
KR700601_A_swine_England_463180_1994_H1N2_1B.1.2
AF085414_A_swine_England_438207_94_H1N2_1B.1
AF085413_A_swine_Scotland_410440_94_H1N2_1B.1
AF085413_A_swine_Scotland_410440_1994_H1N2_1B.1
KU160750_A_swine_North_Carolina_A01945825_2015_H1N2_1B.2.1
KJ437589_A_swine_Oklahoma_A01409770_2014_H1N2_1B.2.1
KT000052_A_swine_North_Carolina_A02077509_2015_H1N2_1B.2.1
KU598293_A_swine_Indiana_A01729197_2016_H1N2_1B.2.1
KU752378_A_swine_North_Carolina_A01730690_2016_H1N2_1B.2.1
HQ896632_A_swine_Indiana_0582_3_2009_H1N2_1B.2.1
CY099151_A_swine_North_Carolina_00839_2005_H1N1_1B.2.1
FJ638298_A_swine_IL_00685_2005_H1N1_1B.2.1

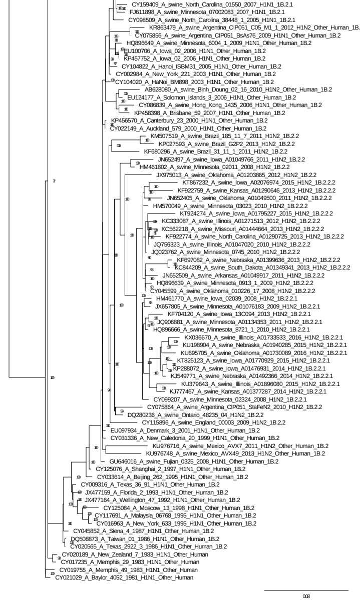

CY159409_A_swine_North_Carolina_01550_2007_H1N1_1B.2.1
FJ611898_A_swine_Minnesota_07002083_2007_H1N1_1B.2.1
CY098509_A_swine_North_Carolina_38448_1_2005_H1N1_1B.2.1
KR863479_A_swine_Argentina_CIP051_C05_M1_1_2012_H1N2_Other_Human_1B.2
CY075856_A_swine_Argentina_CIP051_BsAs76_2009_H1N1_Other_Human_1B.2
HQ896649_A_swine_Minnesota_6004_1_2009_H1N1_Other_Human_1B.2
EU100706_A_Iowa_02_2006_H1N1_Other_Human_1B.2
KP457752_A_Iowa_02_2006_H1N1_Other_Human_1B.2
CY104822_A_Hanoi_ISBM31_2005_H1N1_Other_Human_1B.2
CY002984_A_New_York_221_2003_H1N1_Other_Human_1B.2
CY104020_A_HaNoi_BM898_2003_H1N1_Other_Human_1B.2
AB628080_A_swine_Binh_Doung_02_16_2010_H1N2_Other_Human_1B.2
EU124177_A_Solomon_Islands_3_2006_H1N1_Other_Human_1B.2
CY086839_A_swine_Hong_Kong_1435_2006_H1N1_Other_Human_1B.2
KP458398_A_Brisbane_59_2007_H1N1_Other_Human_1B.2
KP456570_A_Canterbury_23_2000_H1N1_Other_Human_1B.2
CY022149_A_Auckland_579_2000_H1N1_Other_Human_1B.2
KM507519_A_swine_Brazil_185_11_7_2011_H1N2_1B.2.2
KP027593_A_swine_Brazil_G2P2_2013_H1N2_1B.2.2
KF680296_A_swine_Brazil_31_11_1_2011_H1N2_1B.2.2
JN652497_A_swine_Iowa_A01049766_2011_H1N2_1B.2.2
HM461802_A_swine_Minnesota_02011_2008_H1N2_1B.2.2
JX975013_A_swine_Oklahoma_A01203865_2012_H1N2_1B.2.2
KT867232_A_swine_Iowa_A02076974_2015_H1N2_1B.2.2.2
KF922759_A_swine_Kansas_A01290646_2013_H1N2_1B.2.2.2
JN652405_A_swine_Oklahoma_A01049500_2011_H1N2_1B.2.2.2
HM570049_A_swine_Minnesota_03023_2010_H1N2_1B.2.2.2
KT924274_A_swine_Iowa_A01795227_2015_H1N2_1B.2.2.2
KC333087_A_swine_Illinois_A01271513_2012_H1N2_1B.2.2.2
KC562218_A_swine_Missouri_A01444664_2013_H1N2_1B.2.2.2
KF922774_A_swine_North_Carolina_A01290725_2013_H1N2_1B.2.2.2
JQ756323_A_swine_Illinois_A01047020_2010_H1N2_1B.2.2.2
JQ023762_A_swine_Minnesota_0745_2010_H1N2_1B.2.2.2
KF697082_A_swine_Nebraska_A01399636_2013_H1N2_1B.2.2.2
KC844209_A_swine_South_Dakota_A01349341_2013_H1N2_1B.2.2.2
JN652509_A_swine_Arkansas_A01049917_2011_H1N2_1B.2.2.2
HQ896639_A_swine_Minnesota_0913_1_2009_H1N2_1B.2.2.2
CY045599_A_swine_Oklahoma_010226_17_2008_H1N2_1B.2.2.2
HM461770_A_swine_Iowa_02039_2008_H1N2_1B.2.2.1
JX657805_A_swine_Minnesota_A01076183_2009_H1N2_1B.2.2.1
KF704120_A_swine_Iowa_13C094_2013_H1N2_1B.2.2.1
JQ906881_A_swine_Minnesota_A01134353_2011_H1N2_1B.2.2.1
HQ896666_A_swine_Minnesota_8721_1_2010_H1N2_1B.2.2.1
KX036670_A_swine_Illinois_A01733533_2016_H1N2_1B.2.2.1
KU198904_A_swine_Nebraska_A01940285_2015_H1N2_1B.2.2.1
KU695705_A_swine_Oklahoma_A01730089_2016_H1N2_1B.2.2.1
KT825123_A_swine_Iowa_A01770929_2015_H1N2_1B.2.2.1
KP288072_A_swine_Iowa_A01476931_2014_H1N2_1B.2.2.1
KJ549771_A_swine_Nebraska_A01492366_2014_H1N2_1B.2.2.1
KU379643_A_swine_Illinois_A01896080_2015_H1N2_1B.2.2.1
KJ777467_A_swine_Kansas_A01377287_2014_H1N2_1B.2.2.1
CY099207_A_swine_Minnesota_02324_2008_H1N2_1B.2.2.1
CY075864_A_swine_Argentina_CIP051_StaFeN2_2010_H1N2_1B.2.2
DQ280236_A_swine_Ontario_48235_04_H1N2_1B.2.2
CY115896_A_swine_England_00003_2009_H1N2_1B.2.2
EU097934_A_Denmark_3_2001_H1N1_Other_Human_1B.2
CY031336_A_New_Caledonia_20_1999_H1N1_Other_Human_1B.2
KU976716_A_swine_Mexico_AVX7_2011_H1N2_Other_Human_1B.2
KU976748_A_swine_Mexico_AVX49_2013_H1N2_Other_Human_1B.2
GU646016_A_swine_Fujian_0325_2008_H1N1_Other_Human_1B.2
CY125076_A_Shanghai_2_1997_H1N1_Other_Human_1B.2
CY033614_A_Beijing_262_1995_H1N1_Other_Human_1B.2
CY009316_A_Texas_36_91_H1N1_Other_Human_1B.2
JX477159_A_Florida_2_1993_H1N1_Other_Human_1B.2
JX477164_A_Wellington_47_1992_H1N1_Other_Human_1B.2
CY125084_A_Moscow_13_1998_H1N1_Other_Human_1B.2
CY117691_A_Malaysia_06768_1995_H1N1_Other_Human_1B.2
CY016963_A_New_York_633_1995_H1N1_Other_Human_1B.2
CY045852_A_Siena_4_1987_H1N1_Other_Human_1B.2
DQ508873_A_Taiwan_01_1986_H1N1_Other_Human_1B.2
CY020565_A_Texas_2922_3_1986_H1N1_Other_Human_1B.2
CY020189_A_New_Zealand_7_1983_H1N1_Other_Human
CY017235_A_Memphis_29_1983_H1N1_Other_Human
CY019755_A_Memphis_49_1983_H1N1_Other_Human
CY021029_A_Baylor_4052_1981_H1N1_Other_Human
